# Mining of important genetic loci and evaluation of genetic effects for growth traits in Baicheng You Chicken

**DOI:** 10.1016/j.psj.2026.107063

**Published:** 2026-05-01

**Authors:** Tinghao Jiang, Gaoyun You, Haiying Li, Zhuocheng Hou, Xiaoyu Zhao, Wei Dong, Herong Liao, Shihao Zhang, Zhongtao Yin

**Affiliations:** a College of Animal Science, Xinjiang Agricultural University, Urumqi, Xinjiang 830000, China; b College of Animal Science and Technology, China Agricultural University, Haidian, Beijing 100193, China; c Xinjiang Nuqibai City You Chicken Development Co., LTD., Aksu, Xinjiang 842300, China

**Keywords:** Baicheng You Chicken, Growth performance, Whole-genome resequencing, Genome-wide association study

## Abstract

The Baicheng You Chicken is a precious indigenous breed in Xinjiang, China, prized for its strong disease and stress resistance and superior meat quality. However, the lack of scientific breeding and conservation has led to poor production performance, particularly in growth traits. In this study, we collected phenotypic and whole-genome resequencing data from 1,535 18-week-old Baicheng You Chickens (180 males and 1,355 females). After stringent quality control (SNP call rate > 95%, minor allele frequency > 1%), we constructed the breed’s first comprehensive SNP-based genome-wide variation map, which comprised 2,020,743 high-quality SNPs across the genome. The filtered SNPs had high mapping quality (99.73% mapped to the bGalGal1.mat.broiler.GRCg7b reference genome, Q30 = 93.26%) and a reasonable Ti/Tv ratio (2.596), guaranteeing the reliability of subsequent analyses. We estimated genetic effects (SNP-based heritability and phenotypic variance explained (PVE) by individual loci) via the restricted maximum likelihood (REML) method, and performed a genome-wide association study (GWAS) using a mixed linear model (MLM) — with sex as a fixed effect and principal components to correct for population stratification — to identify significant loci and their effect sizes (Beta). All eight growth traits showed moderate to high heritability: body weight (BW) had the highest heritability (0.86±0.11), while chest width (CW, 0.41±0.08) and body slanting length (BSL, 0.43±0.09) were the lowest; keel length (KL), chest girth (CG), pelvic width (PW), chest depth (CD) and shank length (SL) had heritabilities of 0.50±0.09, 0.46±0.09, 0.54±0.09, 0.67±0.10 and 0.74±0.10, respectively. GWAS identified 145 significant SNPs, with a maximum Beta value of 0.39 and PVE ranging from 1.25% to 6.25%. We annotated 22 candidate genes, with *TAPT1, IGF2BP1, ADGRB3, LDB2, NCAPG* and *LCORL* as key candidates. These quantifiable genetic markers and effect estimates provide direct targets for marker-assisted selection (MAS) and valuable resources for future genomic selection (GS) programs, offering a practical approach to improve the breed’s slow growth while preserving its unique meat quality.

## Introduction

Chickens are among the most economically valuable animals worldwide. While intensive breeding in commercial lines has achieved remarkable growth performance, this has often been accompanied by a decline in genetic diversity and the loss of unique traits found in indigenous breeds. Among these, the Baicheng You Chicken stands out as a high-quality local protected breed from Xinjiang, China, renowned for its superior meat quality, distinctive taste, and strong disease resistance. The breed is characterized by a compact, rectangular body shape, black feathers, a single comb, and black beak and shanks. Roosters exhibit brilliant golden hackle feathers and erect, black tail feathers, whereas hens often display a small crest and a well-developed tail fan, with pronounced subcutaneous fat deposition. However, its commercial development is severely constrained by a slow growth rate, low adult body weight, and inefficient feed conversion ratio. For instance, the average adult body weight of Baicheng You Chicken is only approximately 2.4 kg for males and 1.9 kg for females (Wang et al., 2023), which is substantially lower than that of commercial broilers (Wegner et al., 2023). Therefore, unlocking the genetic potential of this underutilized breed to improve its growth performance while preserving its premium meat quality represents a critical goal for sustainable utilization.

Deciphering the genetic basis of growth traits is fundamental for effective genetic improvement. The genetic architecture of these traits in chickens is highly complex (Yang et al., 2021), with thousands of quantitative trait loci (QTLs) documented in databases (Hu et al., 2022). However, the transferability of QTLs and associated markers across genetically distinct breeds, especially from commercial to unimproved indigenous breeds, is often limited due to differences in allele frequencies, linkage disequilibrium patterns, and population-specific genetic backgrounds. Marker-assisted selection (MAS) and genomic selection (GS) have revolutionized poultry breeding by enabling precise selection based on genome-wide markers (Tavárez and Solis de los Santos, 2016). However, the successful application of these strategies in a specific breed like the Baicheng You Chicken first requires the systematic identification of breed-specific or breed-relevant genetic markers and key genes.

Genome-wide association studies (GWAS) powered by whole-genome resequencing (WGRS) offer a powerful solution. Based on a known reference genome, WGRS sequences the entire genome of an individual to detect variation information (Yang et al., 2025) and has been successfully applied to correlate traits with genetic variants (Dehghan, 2018; Ding et al., 2022; Manzanilla-Pech et al., 2022; Zhao et al., 2021). Compared to the SNP arrays commonly used in previous livestock GWAS due to cost constraints (Li et al., 2025), WGRS provides a more comprehensive variant detection, capturing rare and novel polymorphisms. This is particularly advantageous for indigenous breeds like the Baicheng You Chicken, which have not undergone scientific breeding and may harbor unique genetic variants underlying their distinct phenotype. Despite the proven utility of WGRS-GWAS in other breeds, a comprehensive genetic dissection of growth traits using this approach has not yet been conducted for the Baicheng You Chicken.

We hypothesized that the slow growth of Baicheng You Chicken is associated with a distinct spectrum of genetic variants. To test this hypothesis, we designed a study that moves from genetic parameter estimation to marker discovery and application evaluation. we performed WGRS on 1,535 Baicheng You Chickens. We then conducted a high-resolution GWAS to identify genomic regions and candidate genes associated with eight growth and body measurement traits: Body eight (BW), Body slanting length (BSL), Keel length (KL), Chest girth (CG), Pelvic width (PW), Chest width (CW), Chest depth (CD), and Shank length (SL). This study aims to (1) construct the first comprehensive variation map for this breed, (2) estimate the genetic parameters of its growth traits, (3) pinpoint significant SNPs, estimate their effects, and characterize the distribution of these effects (e.g., identifying potential major vs. minor loci), and (4) annotate candidate genes. The quantifiable genetic markers and effect estimates identified here are intended to provide direct targets for MAS and valuable resources for future GS programs, offering a scientific basis for improving the breed's productivity while conserving its unique genetic heritage.

## Materials and methods

### Ethical statement

All experimental procedures involving animals were approved by the Animal Welfare and Ethics Committee of Xinjiang Agricultural University, Urumqi, Xinjiang, China (Approval No. 2023007).

### Experimental animals and phenotypic measurement

The Baicheng You Chickens used in this study (Supplementary Fig. 1) were obtained from the National Baicheng You Chicken Conservation Farm in Baicheng County, Aksu Prefecture, Xinjiang, and were housed in three-tiered cages. All individuals were sampled at 18 weeks of age, which is the typical market age for local Baicheng You Chickens and close to sexual maturity, enabling the assessment of near-adult growth performance. During sampling, roosters were selected as potential sires for future breeding lines based on their morphological characteristics and growth performance, while hens were chosen from the production flock that met the breed's standard morphological criteria. A total of 1,535 healthy individuals were included, consisting of 180 roosters and 1,355 hens. Throughout the rearing period, the chickens had ad libitum access to feed and water (diet composition and nutrient levels are shown in Supplementary Table 1) and followed routine vaccination protocols. On the day the chickens reached 18 weeks of age, blood collection, body weight measurement, and body size measurement were performed. Blood samples were collected via venipuncture into anticoagulant tubes and stored at −20°C for subsequent genomic DNA extraction. Meanwhile, environmental control was implemented for Baicheng You Chickens during the brooding period (1-6 weeks of age) and growing period (7∼18 weeks of age): the core of the brooding period was to match dietary metabolizable energy, adopt "whole-house heating + local heat preservation" to gradually reduce the temperature to 20∼25°C, adjust the relative humidity from 65%∼70% to 55%∼60%, gradually reduce the light duration to 10∼16 hours (light intensity 8∼10Lux), and increase ventilation frequency with age; the growing period aimed to cultivate robust replacement hens, maintain a stable temperature of 18∼24°C, relative humidity of 50%∼60%, weak light of 8-10 hours, and balance ventilation with air exchange and temperature-humidity control to lay a solid foundation for the laying period.

To minimize inter-observer variation and ensure maximal consistency, all phenotypic measurements were performed by a single, highly trained technician following a detailed, pre-established protocol. All measuring instruments were calibrated against certified reference standards prior to the commencement of data collection to ensure measurement accuracy. A total of eight growth and body conformation traits were measured according to standardized protocols as follows:

Body weight (BW): Birds were fasted for 12 hours (overnight) prior to measurement. BW was determined using an electronic balance (accuracy: 0.01 g; model JE3002, Shanghai Puchun Measuring Instrument Co., Ltd., China).

Body slant length (BSL): BSL was measured as the straight-line distance from the anterior edge of the shoulder joint to the posterior edge of the ischial tuberosity using a flexible measuring tape (accuracy: 0.01 mm; Shanghai M&G Stationery Co., Ltd., China).

Keel length (KL): KL was measured as the curved distance along the midline of the sternum from the anterior tip to the posterior end of the keel using a flexible measuring tape.

Chest girth (CG): CG was measured as the horizontal circumference around the body at the widest part of the sternum, behind the wings, using a flexible measuring tape.

Pelvic width (PW): PW was measured as the maximum width between the outer edges of the two ischial tuberosities using digital calipers (accuracy: 0.01 mm; Shanghai Jiuliang Hardware Tools Co., Ltd., China).

Chest width (CW): CW was measured as the maximum width between the outer edges of the two pectoral muscles using digital calipers.

Chest depth (CD): CD was measured as the vertical distance along the body's longitudinal axis from the ventral surface of the sternal keel to the corresponding point on the back (typically at the level of the last thoracic vertebra) using digital calipers.

Shank length (SL): SL was measured as the straight-line distance from the upper edge of the hock joint to the base of the footpad using digital calipers.

To reduce the influence of potential measurement errors or clinically abnormal individuals on the association analysis, data points deviating by more than three standard deviations from the mean were excluded, a common and conservative threshold used in livestock genomic studies to balance data quality and the retention of natural biological variation.

### Sequencing, quality control, and annotation

At 18 weeks of age, 5 mL of blood was collected from each experimental chicken via the subwing vein and stored in EDTA anticoagulant tubes. Genomic DNA was extracted using a commercial kit (Tiangen Biotech Co., Ltd., Beijing, China). The degradation level of DNA was analyzed by 0.8% agarose gel electrophoresis, and the DNA concentration was accurately quantified using a Qubit 3.0 Fluorometer (Thermo Fisher Scientific, USA). Only DNA samples with a total amount exceeding 1.5 μg were selected for library construction.

Qualified DNA samples were used for paired-end (PE) library construction. Libraries were constructed using a standard Illumina-compatible protocol with the NEBNext Ultra II DNA Library Prep Kit (New England Biolabs, USA) to generate fragments with an average insert size of 350 bp. The multiplexed libraries were sequenced on an Illumina NovaSeq 6000 platform (Illumina, San Diego, CA, USA) at Novogene Co., Ltd. (Beijing, China), generating 150-bp paired-end (PE150) reads with an average sequencing depth of approximately 5 × per individual. Stringent quality control measures were implemented throughout the sample preparation and sequencing processes to ensure the final data met the standard.

The raw sequencing reads were first aligned to the bGalGal1.mat.broiler.GRCg7b reference genome using the BWA-MEM algorithm, followed by sorting with sentieon util sort. Subsequently, variant calling for BAM files was performed using Sentieon Haplotyper to generate single-sample variant files in GVCF format. The GVCFtyper module was then used for joint variant calling of multi-sample GVCF files, yielding multi-sample VCF files. To ensure the accuracy of subsequent genetic analyses, quality control (QC) of SNP data was conducted using PLINK (v1.90) with the following criteria: SNPs with a missing genotype rate > 5% (–geno 0.05) and a minor allele frequency (MAF) < 1% (–maf 0.01) were excluded, and samples with a missing rate > 20% (–mind 0.2) were filtered out. Insertion and deletion (INDEL) variants were subjected to the same missing genotype rate filter (–geno 0.05). Given the focus of the current genome-wide association study (GWAS) on single nucleotide polymorphisms, INDELs and other structural variants were excluded from subsequent association analyses.

### Linkage disequilibrium

To obtain independent variant loci, linkage disequilibrium pruning was performed using PLINK (v1.90) with the parameter –indep-pairwise 50 5 0.2, which served to reduce redundancy and improve statistical power. Since the samples used were a subset of the original genotype dataset, and genome-wide association study (GWAS) requires the sample size and order in phenotypic and genotypic files to be completely consistent, sample screening and reordering were conducted. To ensure the integrity and accuracy of genotype data and avoid SNP quality degradation caused by sample size variation, an additional round of QC was implemented using PLINK (v1.90) with the criteria –geno 0.05 and –maf 0.01. Finally, the pruned and quality-controlled SNP set was used for subsequent principal component analysis and as the genotype input for the mixed linear model in the genome-wide association study.

### Principal component analysis

Based on all autosomal SNPs, principal component analysis (PCA) was performed using PLINK (v1.90) to calculate the first 20 principal components (PCs) for each individual. PCA plots were generated with PC1, PC2, and PC3 designated as the x-axis, y-axis, and z-axis, respectively. The significance test of PCs was conducted using EIGENSTRAT software (v6.1.4). Finally, the first 10 PCs were extracted as fixed-effect covariates to correct for the population stratification effect.

### Statistics and analysis

Prior to data analysis, all phenotypic data were rigorously filtered, with only values within the range of mean ± 3 standard deviations (SD) retained. The normality of all phenotypic traits was verified using the Shapiro-Wilk test. For phenotypic data that deviated from the normal distribution, rank-based inverse normal transformation was applied for rescaling to meet the assumptions of subsequent statistical analyses.

### Phenotypic correction and genetic parameter estimation

Genetic parameters for growth traits were estimated using a linear mixed model implemented in the ASReml-R software package (v4.1.0.176). This model was fitted within the Genomic Best Linear Unbiased Prediction (GBLUP) framework, which utilizes genome-wide SNPs to construct the genomic relationship matrix (GRM), thereby capturing the cumulative effect of all SNPs for accurate genetic parameter estimation. The model was specified as follows:

$$y=\text{Xb}+Z_{1}a+Z_{2}c+e$$

In this model: y is the vector of observed phenotypic values.

X is the design matrix for fixed effects, which included sex (binary: male/female) and cage row (to correct for environmental variation such as gradients in light intensity and temperature across cage positions). b is the vector of solutions for the fixed effects.

Z_1_ and Z_2_ are design matrices for the random effects. a is the vector of additive genetic effects, distributed as a∼N(0,G$\sigma_{a}^{2}$), where G is the genomic relationship matrix (GRM) derived from genome-wide SNP data, and $\sigma_{a}^{2}$ is the additive genetic variance. c is the vector of permanent environmental effects, distributed as c∼N(0,I$\sigma_{c}^{2}$), where I is an identity matrix and $\sigma_{c}^{2}$is the variance component for the permanent environmental effect. e is the vector of residual effects, assumed to follow e∼N(0,I$\sigma_{e}^{2}$).

The SNP-based heritability ($h_{SNP}^{2}$)—representing the proportion of phenotypic variance explained by the cumulative effect of all genome-wide SNPs—was calculated as: $h_{SNP}^{2}=\frac{\sigma_{a}^{2}}{\sigma_{a}^{2}{+\sigma }_{c}^{2}{+\sigma }_{e}^{2}}$

Additionally, the proportion of phenotypic variance explained (PVE) by individual significant SNPs was calculated using the formula:

$$\text{PVE}=2\times \text{MAF}\times \left(1-\text{MAF}\right)\times \beta^{2}$$

where MAF is the minor allele frequency of the SNP, and β is the additive effect size estimated from the GWAS model.

This approach allowed for robust estimation of heritability and genetic correlations while accounting for genomic relationships, environmental confounders, and the genetic architecture of growth traits in the Baicheng You Chicken.

### Genome-wide association study

For growth‑related traits, we used GEMMA software (version 0.96, https://github.com/genetics‑statistics/GEMMA/releases, accessed on 17 June 2025) to construct a mixed linear model (MLM) for genome‑wide association analysis. The model incorporated fixed effects to account for non‑genetic and subtle environmental variations, including: Cage row (to correct for minor environmental gradients such as light intensity and temperature across cage positions); Sex (binary: male/female, the primary biological confounder given standardized housing); The first ten principal components (PCs) (derived from genome‑wide SNPs, to correct for population stratification). The MLM was specified as:

y=Xα+Zβ+Wμ+ε

In this model: y represents the vector of observed phenotypic values for the growth trait;

X is the design matrix for fixed effects (cage row, sex, and the first 10 PCs);

α is the vector of estimated fixed‑effect parameters;

Z is the design matrix for single‑nucleotide polymorphism (SNP) effects;

β represents the vector of SNP effect sizes (additive effects);

W is the design matrix for random effects (genomic relationship matrix, GRM, derived from ∼2.02 million high‑quality SNPs to model genetic relatedness);

μ is the vector of predicted random individual genetic effects;

ε denotes the vector of random residuals.

To control for multiple testing, genome‑wide significance thresholds were set using Bonferroni correction. The number of independent tests (N) was determined from the LD‑pruned SNP set generated using PLINK with parameters –indep‑pairwise 50 5 0.2. Based on this pruned set: Genome‑wide significance threshold: p < 4.03 × 10^−8^ (0.05/N); Suggestive significance threshold: p < 8.06 × 10^−7^ (1/N).

This modeling framework ensured that both systematic environmental differences (cage row) and biological/ population‑structure confounders (sex, PCs) were explicitly accounted for, while maintaining statistical power to detect genuine genetic associations.

### Gene functional annotation and enrichment analysis

To evaluate the association between specific loci and unique traits as well as their impacts on phenotypic variation, this study calculated the phenotypic variance explained (PVE) for each significant or suggestively significant SNP locus, The proportion of phenotypic variance explained (PVE) by each significant SNP was calculated using the following formula: PVE = 2 × MAF × (1 - MAF) × β^2^. where MAF represents the minor allele frequency and β denotes the additive effect size. Functional annotation of genes located within ±100 kb of each candidate SNP was performed using the Variant Effect Predictor (VEP) from Ensembl Release 91. Gene Ontology (GO) and Kyoto Encyclopedia of Genes and Genomes (KEGG) enrichment analyses for the candidate genes were subsequently conducted using g:Profiler (version e109_eg56_p17_1d3191d), with statistical significance defined as a false discovery rate (FDR) < 0.05.

### Statistical fine-mapping and colocalization analysis

To pinpoint the most probable causal variants within genome-wide significant loci, we conducted a statistical fine-mapping analysis utilizing the Sum of Single Effects (SuSiE) model. This Bayesian variable selection framework, available in the susieR R package (v0.14.2) (Wang et al., 2020). incorporates local linkage disequilibrium (LD) patterns to compute the posterior inclusion probability (PIP) for each variant, thereby quantifying its causal likelihood. We adopted a uniform prior distribution, assigning an identical initial probability of causality to all variants within each associated locus. From this analysis, we derived 95% credible sets, defined as the minimum collection of variants required to encompass the true causal variant(s) with a cumulative posterior probability of at least 95%.

To functionally interpret the GWAS signals, we integrated our fine-mapping outputs with regulatory quantitative trait loci (QTL) annotations from the chicken GTEx project. We employed a Bayesian colocalization analysis (Giambartolomei et al., 2014) to assess whether distinct traits (e.g., growth phenotypes and gene expression) shared a common causal variant within the same genomic region. Colocalization was considered strongly supported when the posterior probability (PP4) exceeded 0.80. Finally, variants that were members of the SuSiE-derived credible sets andexhibited robust colocalization evidence with regulatory QTLs were designated as high-confidence candidate causal variants for downstream functional validation.

## Results

### Phenotypic statistics

Descriptive statistics for growth traits (body weight and seven body measurements) of Baicheng You Chickens, based on a total of 1,535 individuals (180 males and 1,355 females), are presented in Table 1, Table 2. The coefficient of variation (CV) was utilized to assess phenotypic variation within each sex. In males, the CV ranged from 3.66% (body slanting length, BSL) to 4.78% (chest width, CW), indicating relatively low and stable phenotypic variation. In contrast, females exhibited a broader spectrum of variation, with CV values spanning from 4.92% (BSL) to 11.42% (body weight, BW). Notably, the CV for BW in females was markedly higher than that in males (11.42% vs. 4.04%), implying that female growth trajectories may be more sensitive to environmental factors or genetic diversity.

**Table 1:** Descriptive Statistics of Growth Traits in Male Baicheng You Chickens.

Trait	Count	Mean	Std	CV,%	Min	Max
BW,g	160	2288.05,g	92.43	4.04,%	2166.27,g	2534.57,g
BSL,cm	169	23.34,cm	0.85	3.66,%	21.40,cm	24.90,cm
KL,cm	175	13.57,cm	0.58	4.31,%	12.00,cm	14.80,cm
CG,cm	177	33.13,cm	1.43	4.32,%	29.00,cm	36.80,cm
PW,cm	178	9.78,cm	0.40	4.12,%	8.72,cm	10.79,cm
CW,cm	179	8.10,cm	0.39	4.78,%	6.98,cm	9.16,cm
CD,cm	173	13.23,cm	0.53	4.01,%	10.33,cm	14.19,cm
SL,cm	167	10.17,cm	0.39	3.80,%	9.19,cm	11.07,cm

Note: Count represents the sample size, Mean the mean value, Std the standard deviation, CV the coefficient of variation, Min the minimum value, and Max the maximum value.

**Table 2:** Descriptive Statistics of Growth Traits in Baicheng You Chicken Hens.

Trait	Count	Mean	Std	CV,%	Min	Max
BW,g	1355	1505.41,g	171.94	11.42,%	1098.65,g	2197.45,g
BSL,cm	1353	20.70,cm	1.02	4.92,%	17.50,cm	25.10,cm
KL,cm	1351	11.17,cm	0.78	6.94,%	8.30,cm	14.40,cm
CG,cm	1348	28.55,cm	1.60	5.60,%	22.30,cm	35.90,cm
PW,cm	1351	8.49,cm	0.53	6.30,%	6.53,cm	10.32,cm
CW,cm	1350	7.34,cm	0.50	6.81,%	5.81,cm	8.88,cm
CD,cm	1350	11.05,cm	0.61	5.56,%	9.14,cm	13.38,cm
SL,cm	1355	8.34,cm	0.61	7.32,%	7.08,cm	10.87,cm

Note: Count represents the sample size, Mean the mean value, Std the standard deviation, CV the coefficient of variation, Min the minimum value, and Max the maximum value.

To quantitatively evaluate sexual dimorphism, statistical comparisons were conducted as shown in Table 3. Independent samples t-tests confirmed that all eight growth traits exhibited significant mean differences between sexes (*P* < 0.05), with males consistently demonstrating higher phenotypic values across all measurements (e.g., BW: 2288.05 ± 92.43 g vs. 1505.41 ± 171.94 g). Furthermore, F-test (Levene’s tests) were performed to assess variance homogeneity. The results indicated that six traits—BW, BSL, KL, PW, SL, and CW—exhibited heterogeneous variances (*P* < 0.05), corroborating the CV discrepancies observed in Table 1, Table 2. Conversely, CG and CD showed homogeneous variances (*P* ≥ 0.05). These statistical comparisons confirm that the phenotypic disparities between males and females are not merely descriptive but are statistically robust.

**Table 3:** Baicheng You Chicken Growth Traits: Sexual Dimorphism and Variance Homogeneity Test.

Traits	Mean±STD		CV,%		*P* value	
	Male	Female	Male	Female	t-test	F-test
BW,g	2288.05,g ± 92.43	1505.41,g ± 171.94	4.04,%	11.42,%	<0.05	<0.05
BSL,cm	23.34,cm±0.86	20.7,cm±1.02	3.66,%	4.92,%	<0.05	<0.05
KL,cm	13.57,cm±0.58	11.17,cm±0.78	4.31,%	6.94,%	<0.05	<0.05
CG,cm	33.13,cm±1.43	28.55,cm±1.6	4.32,%	5.60,%	<0.05	=0.32
PW,cm	9.78,cm±0.4	8.49,cm±0.53	4.12,%	6.30,%	<0.05	<0.05
CW,cm	8.1,cm±0.39	7.34,cm±0.5	4.78,%	6.81,%	<0.05	<0.05
CD,cm	13.23,cm±0.53	11.05,cm±0.61	4.01,%	5.56,%	<0.05	=0.40
SL,cm	10.17,cm±0.39	8.34,cm±0.61	3.80,%	7.32,%	<0.05	<0.05

Note: Data are presented as mean ± standard deviation (SD). CV = (SD/Mean) × 100% (coefficient of variation); Variance homogeneity was tested using Levene’s test (F-test), and mean differences between males and females were evaluated using independent samples t-test; Significance was defined as *P* < 0.05; Homogeneous indicates equal variances between sexes (F-test *P* ≥ 0.05), while Heterogeneous indicates unequal variances (F-test *P* < 0.05).

### Genetic parameters and correlation analysis of growth traits

**Fig. 1:**
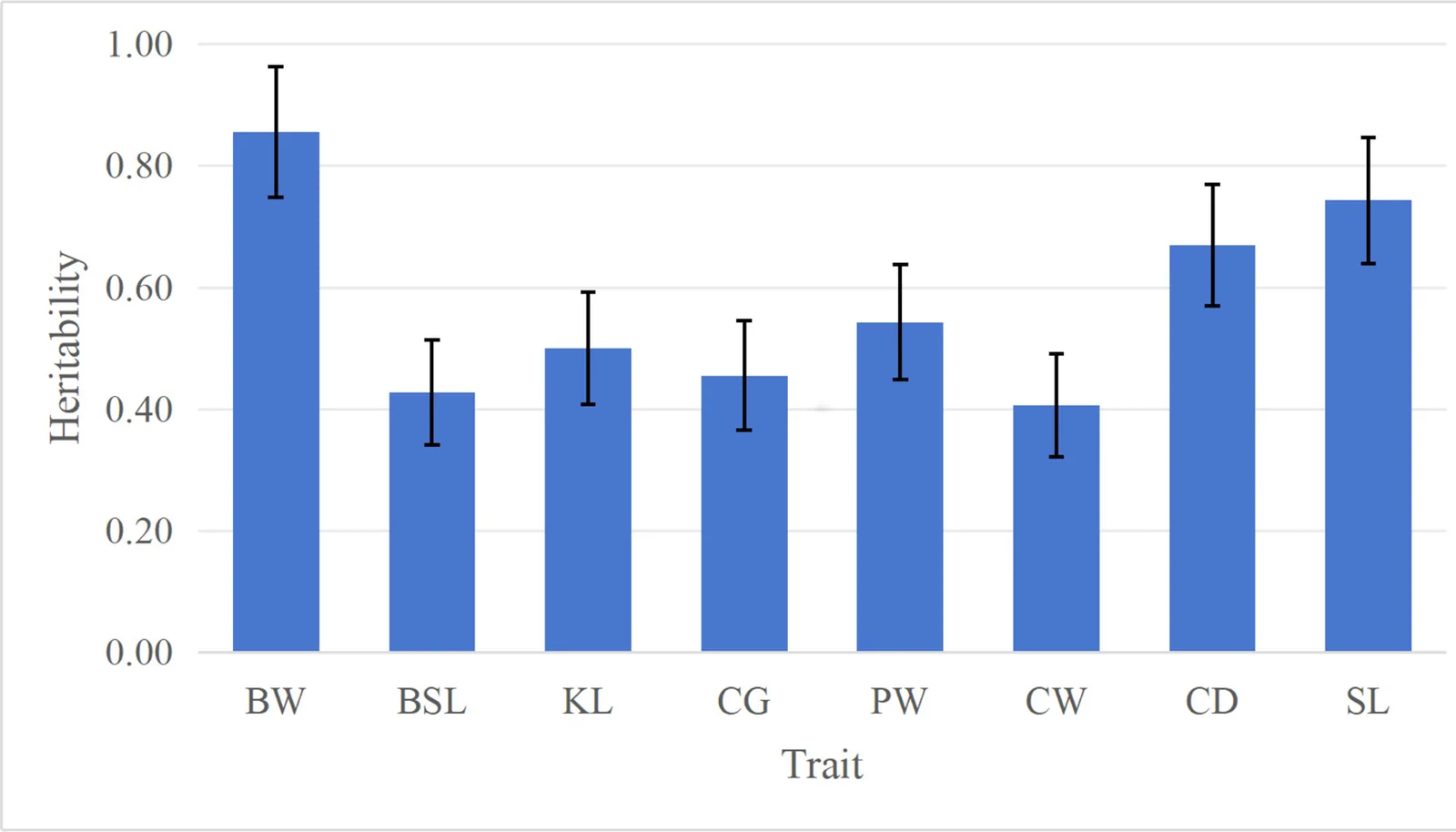
SNP-based Genetic Parameter Estimation. The Y-axis represents SNP heritability, and the X-axis represents phenotypes. Error bars represent the standard error (SE) of the estimates. The higher the heritability, the greater the genetic contribution to the trait.

The genetic architecture and phenotypic relationships of growth traits in Baicheng You Chickens were systematically evaluated using SNP-based methods. Genetic parameter estimation revealed that all eight growth traits exhibited moderate to high heritability, indicating a strong genetic basis. As visualized in Fig. 1, body weight (BW, 0.86 ± 0.11) showed the highest heritability, followed by chest depth (CD, 0.67 ± 0.10) and shank length (SL, 0.74 ± 0.10). Six traits—BW, keel length (KL), chest girth (CG), pelvic width (PW), CD, and SL—exceeded the 0.5 threshold for moderate heritability. Even traits with relatively lower estimates, such as body slanting length (BSL, 0.43) and chest width (CW, 0.41), demonstrated substantial genetic contributions, suggesting these traits are suitable candidates for genomic selection.

To elucidate the relationships between economically important traits, correlation analysis was performed (Table 4). The phenotypic correlations (upper diagonal) showed that meat performance (represented by BW and body measurements) was significantly correlated with body development indices. BW exhibited extremely significant correlations with all body measurement traits (*P* < 0.01), with the strongest association observed with CD (R = 0.854) and the weakest with CW (R = 0.589). This indicates that body measurements, particularly chest depth, are critical predictors of body weight. Furthermore, genetic correlations (lower diagonal) were consistently high across most trait pairs, ranging from 0.76 (CG-CD) to 0.96 (PW-CW). Notably, the genetic correlation between BSL and KL reached 0.95, and that between BSL and SL was also 0.95, suggesting these traits share common genetic pathways or pleiotropic effects. The congruence between high genetic correlations and moderate-to-high heritability estimates underscores the potential for simultaneous genetic improvement of growth rate and body conformation in this breed.

**Table 4:** Phenotypic Correlations (Above Diagonal) and Genetic Correlations (Below Diagonal) Between Carcass Traits of Baicheng You Chicken.

Traits	BW	BSL	KL	CG	PW	CW	CD	SL
BW	1.00	0.64	0.55	0.70	0.62	0.54	0.66	0.63
BSL	0.85	1.00	0.50	0.56	0.40	0.39	0.45	0.68
KL	0.81	0.95	1.00	0.40	0.58	0.49	0.69	0.42
CG	0.89	0.85	0.80	1.00	0.41	0.39	0.46	0.60
PW	0.86	0.80	0.85	0.79	1.00	0.61	0.68	0.34
CW	0.83	0.82	0.87	0.81	0.96	1.00	0.51	0.38
CD	0.85	0.79	0.93	0.76	0.84	0.82	1.00	0.41
SL	0.81	0.95	0.83	0.84	0.61	0.67	0.71	1.00

Note: The lower triangle represents genetic correlations, and the upper triangle represents phenotypic correlations.

### Variant detection

**Fig. 2:**
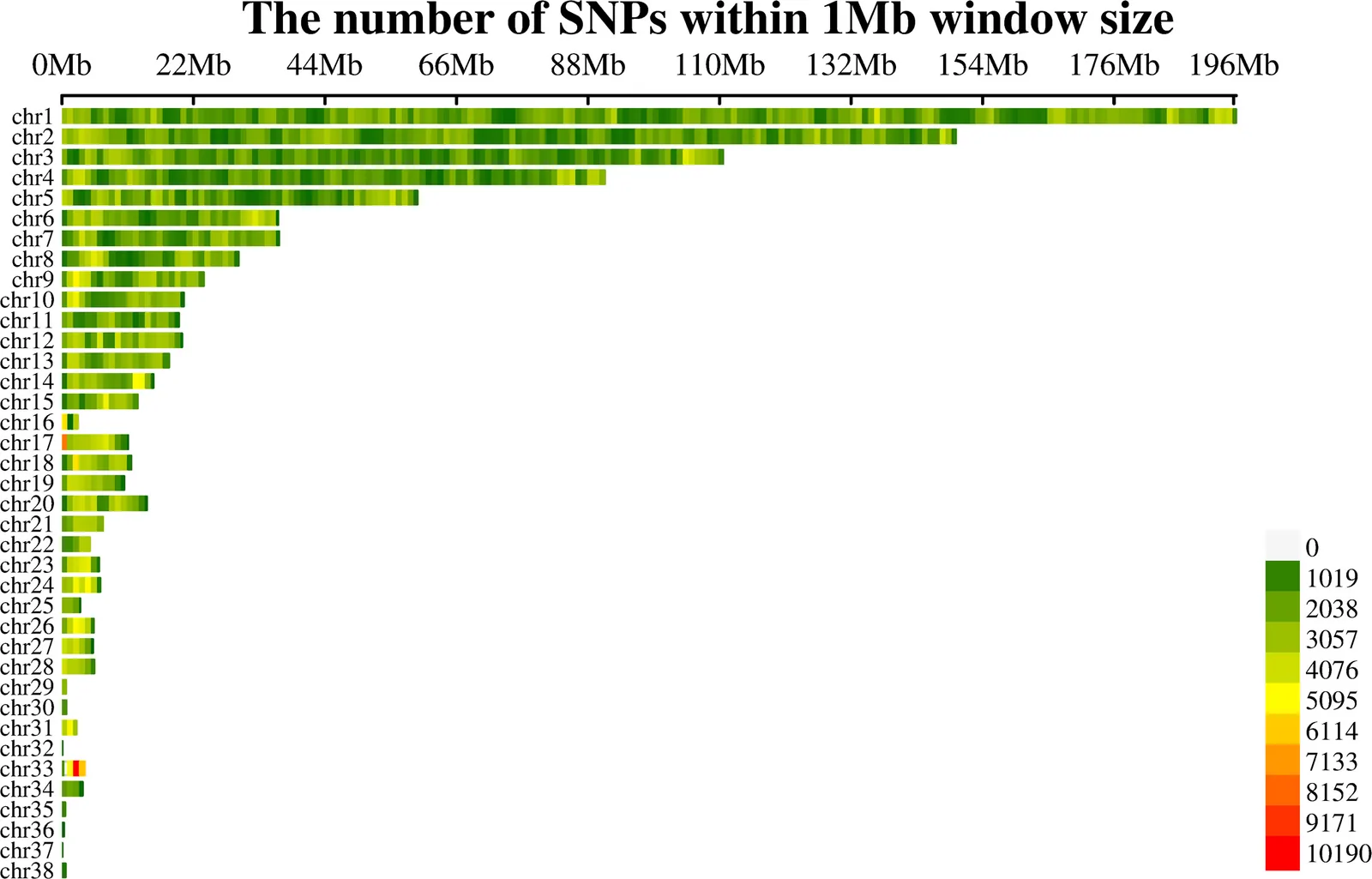
The SNP density map, based on SNPs selected for GWAS analysis, displays SNP densities only for chromosomes 1∼38, encompassing a total of 1986965 SNPs.

Total sequencing yielded 4.94 Tb (avg. 38.02 million PE reads/sample, 5 × depth); 99.73% (99.23∼99.82%) mapped to GRCg7b (Q30=93.26%), with 89.44% properly paired, 0.07% single-end mapped, and Ti/Tv=2.596—confirming high-quality data for variant analysis. GATK identified 28,534,674 variants (25,012,345 SNPs, 3,522,329 INDELs). Stepwise filtering retained 2,020,743 (Fig. 2) SNPs: initial PLINK QC (–geno 0.05/–maf 0.01) removed 12,005,678 SNPs/1,213,277 INDELs; LD pruning (–indep-pairwise 50 5 0.2) removed 6,901,234 SNPs/122,664 INDELs; sample matching (–keep pheno_ids.txt, n = 1,535) reduced SNPs by 10,000; final refinement (second –geno/–maf, –snps-only, –exclude asterisk alleles) removed 25,349/10,563 SNPs and 186,388 INDELs/4,116 SNPs. These SNPs were used for genetic parameter estimation and GWAS.

### Linkage disequilibrium analysis

To describe the genome-wide linkage disequilibrium (LD) patterns, we estimated the LD decay in the Baicheng You chicken population and plotted the relationship between the mean squared correlation coefficient (r²) and physical distance. As shown in Supplementary Fig. 2, the LD decay curve of Baicheng You chickens exhibited a rapid decline with increasing genomic distance. When the r² threshold dropped to 0.2, the LD decay distance was approximately 100 bp.

### Genome-wide association study of growth performance

We first analyzed the population structure of 1,535 Baicheng You Chickens (180 males and 1,355 females). Principal component analysis (PCA) revealed no significant population stratification, indicating the population was well-suited for GWAS. Consistent with this, the genomic inflation factors (λ) for all traits were close to 1 (ranging from 0.9941 to 1.0029), confirming negligible stratification. To further minimize false positives and enhance statistical power, the top 10 principal components (PCs) explaining the major population structure variation were incorporated into the mixed linear model as covariates. Additionally, the first three PCs were visualized in a PCA plot (Supplementary Fig. 3) to characterize and control for residual sample structure.

**Fig. 3:**
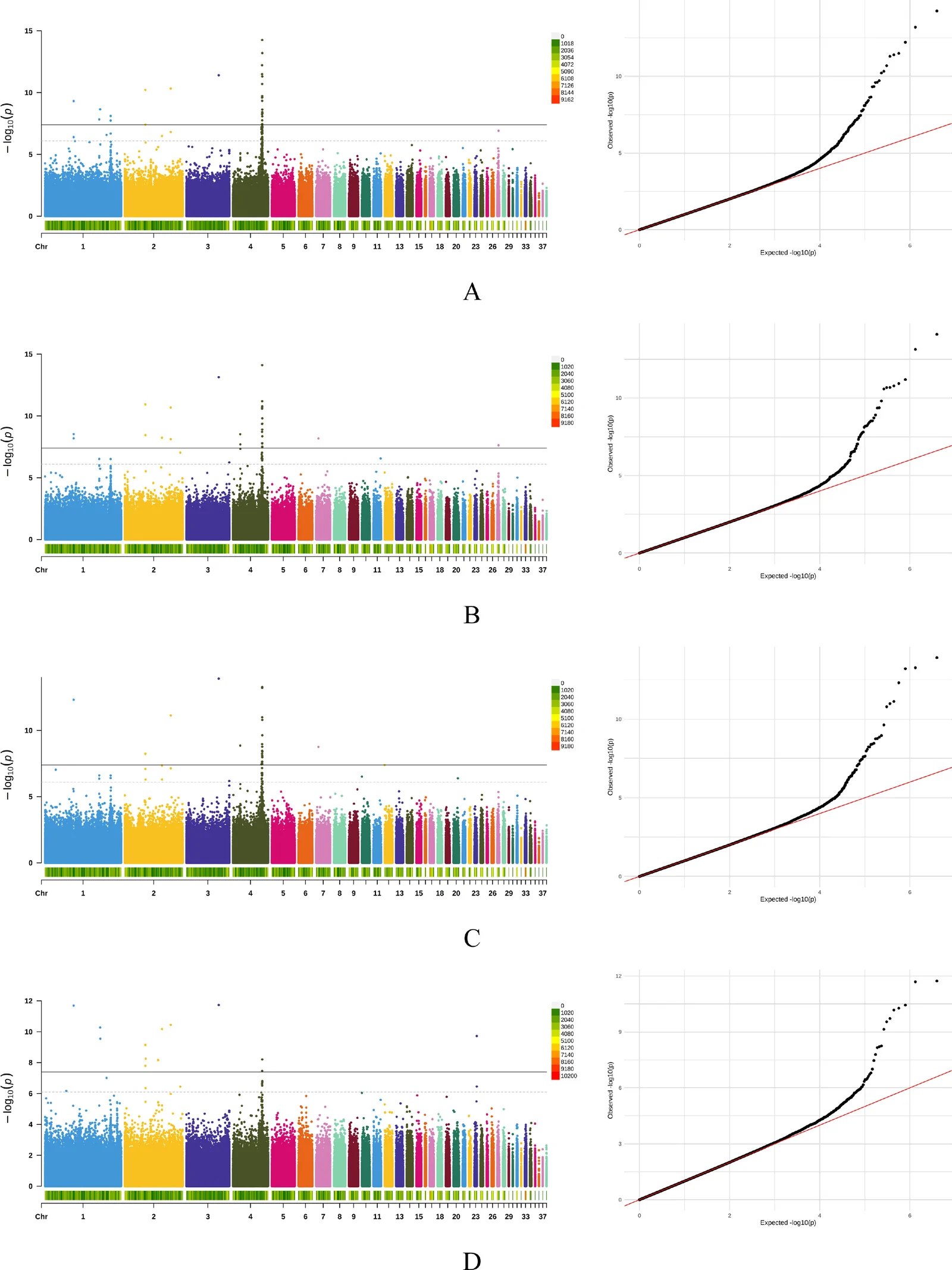

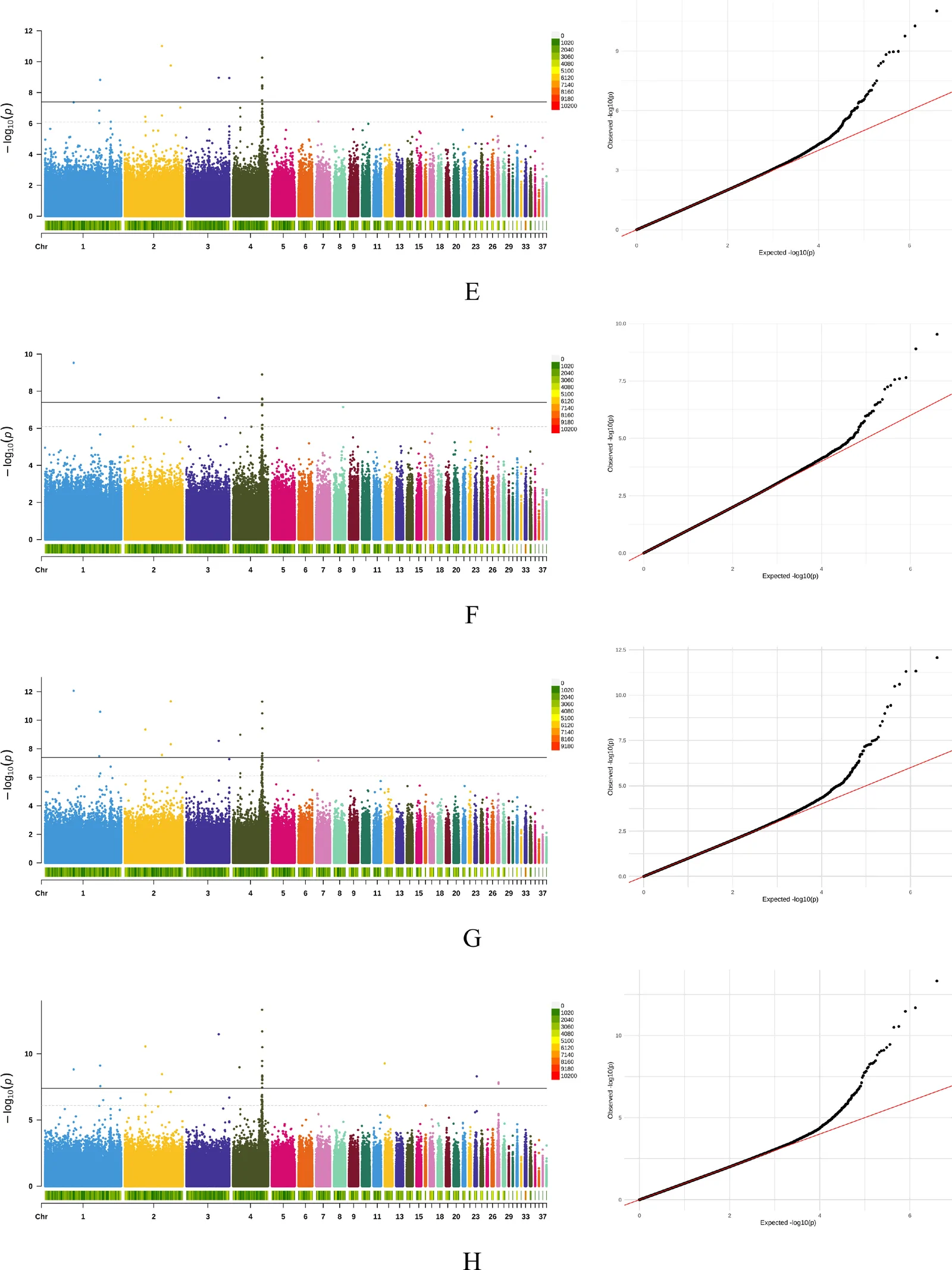
Manhattan plots (left panel) and Quantile-Quantile (Q-Q) plots (right panel) for eight growth traits: (A) Body Weight (BW), (B) Body Slanting Length (BSL), (C) Keel Length (KL), (D) Chest Girth (CG), (E) Pelvic Width (PW), (F) Chest Width (CW), (G) Chest Depth (CD), and (H) Shank Length (SL). The horizontal red dashed line in Manhattan plots indicates the genome-wide significance threshold (*P* < 4.03 × 10^−8^), and the blue dotted line represents the suggestive threshold (*P* < 8.06 × 10^−7^). All traits exhibited λ values close to 1.00 (BSL: 1.0007; BW: 0.9971; CG: 1.0021; CW: 1.0029; KL: 0.9948; PW: 0.9941; CD: 1.0004; SL: 0.9997), indicating negligible genomic inflation and adequate control for population structure and confounding effects.

After SNP filtering, GWAS was performed on 2,020,743 SNPs to identify genomic regions associated with body weight (BW), body slant length (BSL), keel length (KL), chest girth (CG), pelvic width (PW), chest width (CW), chest depth (CD), and shank length (SL). The Manhattan plots and quantile-quantile (Q-Q) plots for each trait are presented in Fig. 3A-H. This analysis identified 145 SNPs with genome-wide significant associations with growth traits at *P* < 4.03 × 10^-8^, as well as 297 SNPs (Detailed data are provided in Supplementary Tables 2) suggestive of significance at *P* < 8.06 × 10^-7^. Information on all significant SNPs is listed in Table 5. These SNPs were distributed across 8 chromosomes (GGA1, GGA2, GGA3, GGA4, GGA7, GGA12, GGA23, and GGA27). Specifically, 27 significant SNPs associated with body weight (BW) were distributed on chromosomes 1, 2, 3, and 4. For body slanting length (BSL) and keel length (KL), 27 and 24 significant SNPs were detected mainly on chromosomes 1∼4 and 7. 13, 11, 5, 15, and 23 significant SNPs were obtained for chest girth (CG), pelvic width (PW), chest width (CW), chest depth (CD), and shank length (SL), respectively, located primarily on chromosomes 1∼4, with a small number on chromosomes 7, 12, 23, and 27. These significant loci were mainly linked to key candidate genes including *KCNA1, ADGRB3, LCORL, NCAPG, LDB2, IGF2BP1*, and *TAPT1*.

**Table 5:** SNPs Associated with Growth Traits in Baicheng You Chickens.

Traits	Chr	Position	MAF	Beta±SE	P-value	PVE (%)	Candidate/Nearst gene
BW	1	73936269	0.256	−0.3 ± 0.05	4.85 × 10^-10^	3.34	KCNA1
BW	1	140034869	0.09	0.36±0.06	1.48 × 10^-8^	2.17	TNFSF13B
BW	1	142237956	0.172	−0.31±0.05	2.25 × 10^-9^	2.76	ENSGALG00010006221
BW	1	169234935	0.443	0.19±0.03	1.83 × 10^-8^	1.85	SPRYD7
BW	1	169338318	0.496	0.19±0.03	7.82 × 10^-9^	1.89	TRIM13
BW	2	53096913	0.117	−0.31±0.06	3.92 × 10^-8^	1.95	CNTNAP2
BW	2	53097194	0.179	−0.32±0.05	6.10 × 10^-11^	3.06	CNTNAP2
BW	2	118607373	0.326	−0.33±0.05	4.74 × 10^-11^	4.84	HNF4G
BW	3	83455455	0.251	−0.33±0.05	4.00 × 10^-12^	4.03	ADGRB3
BW	4	75262244	0.252	−0.22±0.04	5.60 × 10^-9^	1.89	LCORL
BW	4	75283849	0.314	−0.23±0.04	2.56 × 10^-10^	2.21	LCORL
BW	4	75302291	0.393	0.24±0.03	6.08 × 10^-13^	2.86	LCORL
BW	4	75313181	0.355	0.24±0.03	3.22 × 10^-12^	2.63	LCORL
BW	4	75424744	0.437	−0.18±0.03	3.69 × 10^-8^	1.67	LCORL,NCAPG
BW	4	75523761	0.098	0.35±0.06	4.67 × 10^-10^	2.19	QDPR
BW	4	75539579	0.475	0.22±0.03	2.04 × 10^-11^	2.5	QDPR
BW	4	75541018	0.422	0.27±0.03	5.54 × 10^-15^	3.47	QDPR
BW	4	75551305	0.478	−0.19±0.03	1.77 × 10^-8^	1.73	QDPR
BW	4	75581922	0.352	0.19±0.03	3.50 × 10^-8^	1.62	LDB2
BW	4	75645179	0.112	0.33±0.05	1.93 × 10^-10^	2.22	LDB2
BW	4	75650902	0.44	−0.21±0.03	2.46 × 10^-10^	2.15	LDB2
BW	4	75683144	0.501	−0.2 ± 0.03	3.74 × 10^-9^	1.93	LDB2
BW	4	75734711	0.12	0.29±0.05	8.19 × 10^-9^	1.76	LDB2
BW	4	75781424	0.115	0.31±0.05	2.38 × 10^-9^	1.95	LDB2
BW	4	75789407	0.235	0.29±0.04	6.34 × 10^-14^	3.05	LDB2
BW	4	75801513	0.248	0.27±0.04	5.03 × 10^-12^	2.71	LDB2
BW	4	76090544	0.298	−0.21±0.04	4.71 × 10^-9^	1.86	PROM1
BSL	1	73936138	0.054	0.48±0.08	2.97 × 10^-9^	2.39	KCNA1
BSL	1	73936269	0.254	−0.29±0.05	6.50 × 10^-9^	3.14	KCNA1
BSL	2	53096913	0.116	−0.35±0.06	3.56 × 10^-9^	2.46	CNTNAP2
BSL	2	53097194	0.178	−0.35±0.05	1.17 × 10^-11^	3.56	CNTNAP2
BSL	2	95903907	0.34	−0.29±0.05	5.85 × 10^-9^	3.74	MC2R
BSL	2	118607373	0.326	−0.35±0.05	2.11 × 10^-11^	5.4	HNF4G
BSL	2	118607523	0.234	−0.29±0.05	7.69 × 10^-9^	3.01	HNF4G
BSL	3	83455455	0.25	−0.37±0.05	7.37 × 10^-14^	5.07	ADGRB3
BSL	4	18903563	0.193	−0.29±0.05	3.02 × 10^-9^	2.69	SLITRK2
BSL	4	19212102	0.291	0.3 ± 0.05	2.01 × 10^-8^	3.64	TRIM2
BSL	4	75283849	0.313	−0.23±0.04	4.45 × 10^-10^	2.19	LCORL
BSL	4	75302291	0.395	0.23±0.03	2.63 × 10^-11^	2.49	LCORL
BSL	4	75313181	0.359	0.21±0.03	1.93 × 10^-9^	2.01	LCORL
BSL	4	75424744	0.434	−0.23±0.03	2.14 × 10^-11^	2.49	LCORL,NCAPG
BSL	4	75539579	0.479	0.26±0.03	7.87 × 10^-15^	3.36	QDPR
BSL	4	75541018	0.425	0.23±0.03	6.52 × 10^-12^	2.7	QDPR
BSL	4	75551305	0.477	−0.19±0.03	1.59 × 10^-8^	1.76	QDPR
BSL	4	75581922	0.353	0.19±0.03	1.70 × 10^-8^	1.72	LDB2
BSL	4	75645179	0.112	0.32±0.05	1.25 × 10^-9^	2.02	LDB2
BSL	4	75650902	0.439	−0.22±0.03	1.67 × 10^-11^	2.43	LDB2
BSL	4	75664504	0.118	0.28±0.05	3.09 × 10^-8^	1.67	LDB2
BSL	4	75734711	0.121	0.28±0.05	3.37 × 10^-8^	1.64	LDB2
BSL	4	75781424	0.116	0.32±0.05	4.29 × 10^-10^	2.13	LDB2
BSL	4	75789407	0.235	0.25±0.04	1.57 × 10^-10^	2.2	LDB2
BSL	4	75801513	0.25	0.23±0.04	4.46 × 10^-9^	1.98	LDB2
BSL	7	5898011	0.146	−0.31±0.05	6.61 × 10^-9^	2.42	UGT1A1
BSL	27	3286242	0.44	0.19±0.03	2.37 × 10^-8^	1.74	IGF2BP1
KL	1	73936269	0.252	−0.36±0.05	4.81 × 10^-13^	4.77	KCNA1
KL	2	53097194	0.177	−0.3 ± 0.05	5.78 × 10^-9^	2.59	CNTNAP2
KL	2	118607373	0.325	−0.36±0.05	7.45 × 10^-12^	5.56	HNF4G
KL	3	83455455	0.249	−0.38±0.05	1.22 × 10^-14^	5.33	ADGRB3
KL	4	18903563	0.192	−0.3 ± 0.05	1.40 × 10^-9^	2.78	SLITRK2
KL	4	73472843	0.42	−0.19±0.03	3.55 × 10^-8^	1.74	PPARGC1A
KL	4	75283849	0.312	−0.2 ± 0.04	2.48 × 10^-8^	1.77	LCORL
KL	4	75291318	0.196	0.24±0.04	2.43 × 10^-8^	1.75	LCORL
KL	4	75302291	0.394	0.2 ± 0.03	5.77 × 10^-9^	1.91	LCORL
KL	4	75424744	0.434	−0.21±0.03	1.10 × 10^-9^	2.08	LCORL,NCAPG
KL	4	75523761	0.102	0.31±0.06	2.22 × 10^-8^	1.79	QDPR
KL	4	75533549	0.073	0.38±0.06	3.43 × 10^-9^	1.98	QDPR
KL	4	75539579	0.48	0.25±0.03	6.15 × 10^-14^	3.17	QDPR
KL	4	75541018	0.425	0.23±0.03	1.02 × 10^-11^	2.67	QDPR
KL	4	75645179	0.114	0.39±0.05	5.33 × 10^-14^	3.1	LDB2
KL	4	75650902	0.438	−0.19±0.03	8.29 × 10^-9^	1.81	LDB2
KL	4	75664504	0.12	0.31±0.05	1.71 × 10^-9^	1.97	LDB2
KL	4	75683144	0.498	−0.19±0.03	3.36 × 10^-8^	1.72	LDB2
KL	4	75734711	0.122	0.3 ± 0.05	3.79 × 10^-9^	1.87	LDB2
KL	4	75781424	0.118	0.32±0.05	2.33 × 10^-10^	2.2	LDB2
KL	4	75789407	0.237	0.26±0.04	1.62 × 10^-11^	2.45	LDB2
KL	4	75801513	0.251	0.22±0.04	1.07 × 10^-8^	1.89	LDB2
KL	4	76005429	0.215	0.24±0.04	4.05 × 10^-9^	1.99	TAPT1
KL	7	5898011	0.146	−0.32±0.05	1.77 × 10^-9^	2.59	UGT1A1
CG	1	73936269	0.252	−0.35±0.05	2.05 × 10^-12^	4.58	KCNA1
CG	1	142237956	0.17	−0.35±0.05	5.33 × 10^-11^	3.46	ENSGALG00010006221
CG	1	142714957	0.339	−0.32±0.05	2.86 × 10^-10^	4.6	SLC10A2
CG	2	53096913	0.115	−0.33±0.06	1.63 × 10^-8^	2.25	CNTNAP2
CG	2	53097194	0.177	−0.32±0.05	7.23 × 10^-10^	2.94	CNTNAP2
CG	2	54057299	0.479	−0.23±0.04	5.63 × 10^-9^	2.64	PDIA4
CG	2	86041152	0.062	0.43±0.07	6.91 × 10^-9^	2.17	IRX2
CG	2	95903907	0.338	−0.32±0.05	6.70 × 10^-11^	4.69	MC2R
CG	2	118607373	0.324	−0.35±0.05	3.64 × 10^-11^	5.25	HNF4G
CG	3	83455455	0.248	−0.35±0.05	1.86 × 10^-12^	4.48	ADGRB3
CG	4	75541018	0.425	0.19±0.03	3.44 × 10^-8^	1.77	QDPR
CG	4	75650902	0.438	−0.19±0.03	6.26 × 10^-9^	1.86	LDB2
CG	23	6175317	0.184	0.34±0.05	1.91 × 10^-10^	3.49	RCAN3
PW	1	142237956	0.17	−0.32±0.05	1.52 × 10^-9^	2.89	ENSGALG00010006221
PW	2	95903907	0.339	−0.33±0.05	9.65 × 10^-12^	4.98	MC2R
PW	2	118607373	0.324	−0.33±0.05	1.76 × 10^-10^	4.78	HNF4G
PW	3	83455455	0.248	−0.3 ± 0.05	1.10 × 10^-9^	3.3	ADGRB3
PW	3	110589169	0.153	0.32±0.05	1.15 × 10^-9^	2.73	MRPL19
PW	4	75302291	0.395	0.19±0.03	3.16 × 10^-8^	1.72	LCORL
PW	4	75313181	0.36	0.21±0.03	1.05 × 10^-9^	2.05	LCORL
PW	4	75539579	0.48	0.2 ± 0.03	4.14 × 10^-9^	1.95	QDPR
PW	4	75541018	0.425	0.22±0.03	5.49 × 10^-11^	2.47	QDPR
PW	4	75645179	0.113	0.31±0.05	3.39 × 10^-9^	1.91	LDB2
PW	4	75675395	0.285	0.22±0.04	5.50 × 10^-9^	1.92	LDB2
CW	1	73936269	0.253	−0.31±0.05	2.93 × 10^-10^	3.7	KCNA1
CW	3	83455455	0.248	−0.28±0.05	2.25 × 10^-8^	2.85	ADGRB3
CW	4	75313181	0.358	0.21±0.03	1.26 × 10^-9^	2.06	LCORL
CW	4	75539579	0.48	0.19±0.03	2.52 × 10^-8^	1.77	QDPR
CW	4	75581922	0.356	0.19±0.03	2.77 × 10^-8^	1.68	LDB2
CD	1	73936269	0.253	−0.35±0.05	8.58 × 10^-13^	4.52	KCNA1
CD	1	140034869	0.091	0.36±0.06	3.37 × 10^-8^	2.14	TNFSF13B
CD	1	142237956	0.169	−0.35±0.05	2.52 × 10^-11^	3.49	ENSGALG00010006221
CD	2	53097194	0.177	−0.31±0.05	4.43 × 10^-10^	2.86	CNTNAP2
CD	2	95903907	0.34	−0.27±0.05	2.72 × 10^-8^	3.27	MC2R
CD	2	118607373	0.324	−0.35±0.05	4.76 × 10^-12^	5.48	HNF4G
CD	2	118607523	0.233	−0.29±0.05	4.86 × 10^-9^	2.97	HNF4G
CD	3	83455455	0.249	−0.29±0.05	2.80 × 10^-9^	3.08	ADGRB3
CD	4	19076489	0.196	0.3 ± 0.05	1.03 × 10^-9^	2.93	TRIM2
CD	4	75291318	0.196	0.23±0.04	3.24 × 10^-8^	1.72	LCORL
CD	4	75539579	0.479	0.23±0.03	4.96 × 10^-12^	2.67	QDPR
CD	4	75645179	0.112	0.35±0.05	3.27 × 10^-11^	2.42	LDB2
CD	4	75675395	0.285	0.21±0.04	3.07 × 10^-8^	1.73	LDB2
CD	4	75781424	0.117	0.29±0.05	2.09 × 10^-8^	1.72	LDB2
CD	4	75789407	0.236	0.24±0.04	3.71 × 10^-10^	2.13	LDB2
SL	1	73936269	0.254	−0.29±0.05	1.51 × 10^-9^	3.21	KCNA1
SL	1	142237956	0.171	−0.32±0.05	7.91 × 10^-10^	2.94	ENSGALG00010006221
SL	1	142714957	0.339	−0.28±0.05	2.75 × 10^-8^	3.4	SLC10A2
SL	2	53097194	0.178	−0.33±0.05	2.81 × 10^-11^	3.23	CNTNAP2
SL	2	95903907	0.341	−0.28±0.05	3.44 × 10^-9^	3.65	MC2R
SL	3	83455455	0.249	−0.33±0.05	3.39 × 10^-12^	4.14	ADGRB3
SL	4	17036502	0.092	−0.37±0.06	1.05 × 10^-9^	2.28	IL1RAPL2
SL	4	75302291	0.394	0.21±0.03	8.45 × 10^-10^	2.1	LCORL
SL	4	75313181	0.357	0.26±0.03	4.78 × 10^-14^	3.09	LCORL
SL	4	75424744	0.433	−0.19±0.03	1.71 × 10^-8^	1.76	LCORL,NCAPG
SL	4	75523761	0.101	0.35±0.06	3.51 × 10^-10^	2.22	QDPR
SL	4	75539579	0.478	0.23±0.03	2.05 × 10^-12^	2.75	QDPR
SL	4	75541018	0.423	0.19±0.03	3.57 × 10^-8^	1.75	QDPR
SL	4	75581922	0.352	0.2 ± 0.03	5.60 × 10^-9^	1.81	LDB2
SL	4	75645179	0.113	0.3 ± 0.05	8.26 × 10^-9^	1.83	LDB2
SL	4	75650902	0.44	−0.19±0.03	4.34 × 10^-9^	1.87	LDB2
SL	4	75781424	0.117	0.3 ± 0.05	5.10 × 10^-9^	1.87	LDB2
SL	4	75789407	0.235	0.26±0.04	3.17 × 10^-11^	2.39	LDB2
SL	4	75801513	0.249	0.22±0.04	9.81 × 10^-9^	1.89	LDB2
SL	12	854	0.4	−0.35±0.06	5.32 × 10^-10^	6.02	RRP9
SL	23	6175317	0.182	0.31±0.05	5.09 × 10^-9^	2.81	RCAN3
SL	27	3286242	0.439	0.19±0.03	1.49 × 10^-8^	1.75	IGF2BP1
SL	27	3290975	0.447	0.19±0.03	1.85 × 10^-8^	1.76	GIP

Note: This table presents single nucleotide polymorphisms (SNPs) that reached the genome-wide significance level (*P* < 4.45 × 10^−8^) or suggestive significance level (*P* < 8.91 × 10^−7^) in the genome-wide association study (GWAS), following Bonferroni correction for multiple testing. Abbreviations: BW, Body Weight; BSL, Body Slant Length; KL, Keel Length; CG, Chest Girth; PW, Pelvic Width; CW, Chest Width; CD, Chest Depth; SL, Shank Length; Chr, Chromosome Number; Position, Chromosomal Physical Position; MAF, Minor Allele Frequency; Beta±SE, Regression Coefficient ± Standard Error; *P*-value, Association Significance *P*-value (corrected); PVE (%), Phenotypic Variance Explained (%); Candidate/Nearest gene, Candidate/Nearest Gene.

The phenotypic variance explained (PVE) by all SNPs ranged from 1.25% to 6.25%. We used the Variant Effect Predictor (VEP) to annotate the genes potentially regulated by the significantly associated SNPs. To further explore the regulatory effects of intergenic variants on traits and genes, we performed additional annotation of regulatory elements using chicken Genotype-Tissue Expression (GTEx) data. A total of 10 significantly associated SNPs were annotated as enhancer regions in chickens (Supplementary Table 3), suggesting that these variants have potential regulatory effects on gene expression levels. The quantile-quantile plots for all traits were clearly aligned along the diagonal with a slight upward tail, indicating that false positives were effectively controlled and the model was reasonable.

### Function enrichment analyses of identified associated genes

**Fig. 4:**
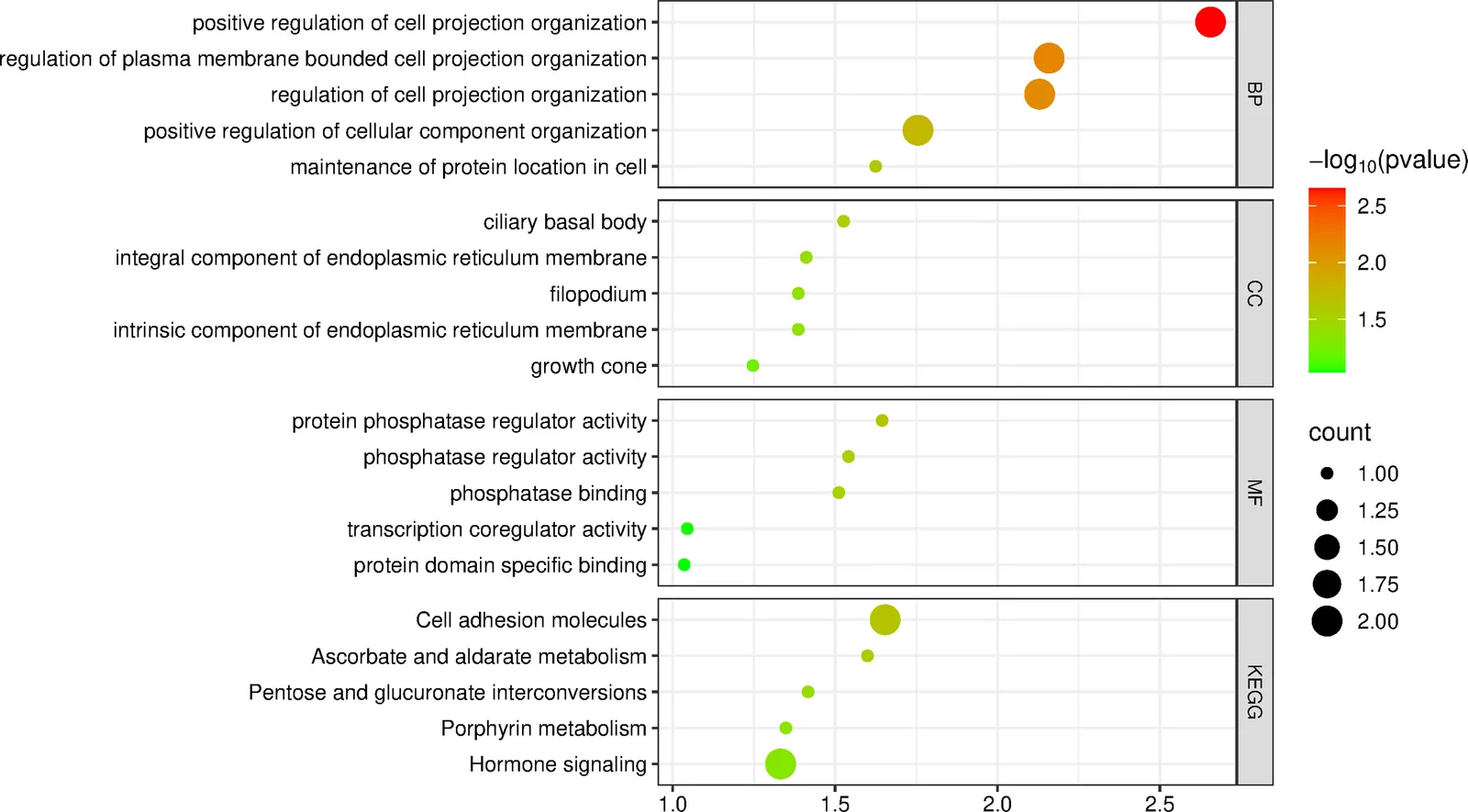
Gene Ontology (GO) and Kyoto Encyclopedia of Genes and Genomes (KEGG) enrichment analyses of candidate genes associated with growth traits in Baicheng You Chicken. The top significantly enriched biological processes, cellular components, molecular functions (GO terms), and signaling pathways (KEGG) are presented. Darker colors indicate more significant enrichment, and larger dots represent a greater number of enriched genes.

A total of 22 candidate genes significantly associated with the growth traits of Baicheng You chickens were identified via genome-wide analysis in this study. To further clarify the potential biological functions of these genes, we performed Gene Ontology (GO) and Kyoto Encyclopedia of Genes and Genomes (KEGG) pathway enrichment analyses, aiming to explore the candidate genes that determine the production performance of Baicheng You chickens. As shown in Fig. 4, the top 5 most significantly enriched terms from Gene Ontology (GO) and Kyoto Encyclopedia of Genes and Genomes (KEGG) analyses are presented. These candidate genes were significantly enriched in key biological processes and pathways including positive regulation of cell projection organization, ciliary basal body, protein phosphatase regulator activity, and cell adhesion molecules.

The findings indicated that *TAPT1*, a gene identified through SNP annotation, was enriched in 71 significant GO terms, including participation in the formation and maintenance of cell protrusions (GO:0031344/0120035/0031346). It may affect the morphological polarization of osteoblasts and myoblasts by regulating the dynamic assembly of microtubules or microfilaments, thereby further influencing skeletal and muscular development. Additionally, it regulates the localization and maintenance of proteins in the endoplasmic reticulum (GO:0032507/0070972/0045185), which might be involved in the synthesis and transport of secreted proteins such as collagens and growth factors, thus affecting the development of connective tissues (e.g., cartilage and bone matrix).

*IGF2BP1* was enriched in 67 significant GO terms, including synergistic inhibition of macromolecule biosynthesis with *LDB2* (GO:2000113/0010558/0031327), participation in the process of cell projection organization (GO:0030030/0120036), and synergistic regulation of cell protrusion formation (GO:0031344/0120035/0031346). The complete results are provided in Supplementary Table 4.

### Fine-mapping for traits

**Fig. 5:**
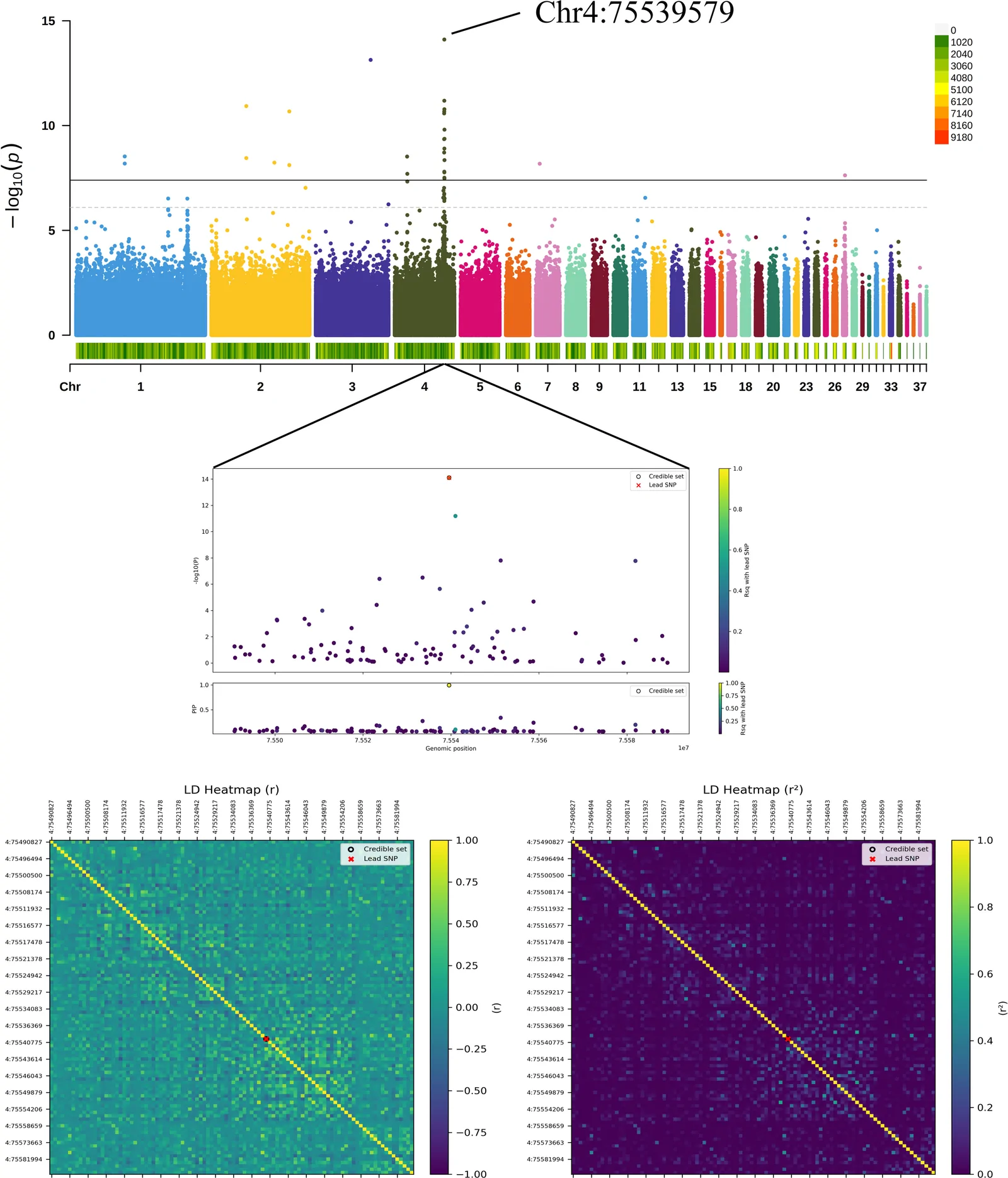
Fine-mapping of locus GGA4:75539579 for body slanting length (BSL) trait.The top panel displays the regional association plot, with each point representing a SNP. The middle panel shows the statistical fine-mapping results using the SuSiE model. The bottom panel presents the linkage disequilibrium (LD) heatmap based on r² values relative to the lead SNP (GGA4:75539579).

For the candidate quantitative trait locus (QTL) regions listed in Table 5, fine-mapping analysis calculated the posterior causal probability (PPC) of each variant and gene, identifying candidate genes consistent with the results of the genome-wide association study (GWAS). A total of 22 genes detected in the GWAS signals were successfully fine-mapped, including *QDPR, ADGRB3, LDB2, MC2R, KCNA1*, and *LCORL*. Among these, the variant at chr4:75539579 (Fig. 5) was significantly associated with body slant length (BSL) and was in a state of strong linkage disequilibrium with the surrounding single nucleotide polymorphisms (SNPs). These results indicated that the variants identified via GWAS were reliable and provide reliable data support for developing molecular markers related to growth performance (Supplementary Fig. 4 for complete results).

## Discussion

The variation in body size among domestic chickens is a typical characteristic of domestication selection and a key determinant of meat production efficiency. However, previous studies relying on low-density genotyping arrays have failed to capture the full spectrum of genetic variants in local breeds (Geibel et al., 2021), resulting in a poor characterization of their genetic architecture. The Baicheng You chicken harbors distinct genetic variations related to growth, yet its regulatory network remains unclear. This study utilizes whole-genome sequencing (WGS) to systematically uncover the genetic architecture of its growth traits, aiming to fill the gap in the detailed genetic mapping of local chicken breeds.

Building upon the phenotypic and population structure analyses, this study presents the first comprehensive estimation of genetic parameters for growth traits in Baicheng You Chicken, utilizing a high-density SNP dataset derived from whole-genome sequencing. The estimated SNP-based heritabilities ranged from 0.41 to 0.86, indicating that these traits are predominantly controlled by additive genetic effects (Moreira et al., 2019) and are thus highly amenable to genomic selection. Notably, body weight (BW) exhibited the highest heritability (0.86 ± 0.11), consistent with previous studies (Kanlisi et al., 2024). underscoring its strong genetic determinism and the high accuracy expected for genomic prediction in this breed. In contrast, chest width (CW) and body slanting length (BSL) showed relatively lower, albeit moderate (Adeleke et al., 2011), heritability estimates (0.41 ± 0.08 and 0.43 ± 0.09, respectively). This suggests that while genetic improvement is feasible, these traits may be more susceptible to environmental variance or involve more complex epistatic interactions compared to BW (Zuk et al., 2012). Consistent with the highly polygenic architecture of complex traits, individual significant SNPs explained only a small proportion of the phenotypic variance (PVE: 1.25∼6.25%). However, the high cumulative heritabilities estimates demonstrate that the collective effect of the numerous variants identified via WGS is substantial. Furthermore, the genetic correlations between traits were remarkably high (0.76∼0.96), confirming that these morphometric traits are regulated by a shared genetic network (Zhang et al., 2021). This finding has profound implications for breeding: the high genetic correlation between BSL, keel length (KL), and shank length (SL) (up to 0.95) implies that selecting for increased body length or shank length would likely yield correlated responses in other body measurements, thereby facilitating the simultaneous genetic improvement of overall body conformation and growth rate in the Baicheng You Chicken.

To elucidate the genetic architecture governing growth traits in Baicheng You Chicken, we performed a GWAS. Given the focus on a well-defined population of indigenous chickens and the high minor allele frequency (MAF > 1%) of the identified variants, the average sequencing depth of ∼5 × per individual was deemed sufficient to ensure accurate genotype calling and robust association signals, consistent with established protocols for population-based GWAS (Pasaniuc et al., 2012). Despite the moderate depth, rigorous QC (call rate >95%, MAF >1%) and high data quality (mapping rate: 99.73%; Q30: 93.46%) ensured reliable genotypes (Hemstrom et al., 2024). Furthermore, genomic inflation factors (λ ≈ 1.00) and QQ plots confirmed negligible population stratification and technical noise in our mixed linear model (Reed et al., 2015). To ensure the rigor of multiple testing correction, we adopted a dual-threshold Bonferroni strategy based on LD-pruned independent SNPs (N = 1240917, via –indep-pairwise 50 5 0.2optimized for Baicheng You Chicken’s LD structure), using a genome-wide significance threshold (P < 4.03 × 10^−8^, 0.05/N) to control family-wise error rate (FWER) at 5% for validating known QTLs and a suggestive threshold (P < 8.06 × 10^−7^, 1/N) for exploring breed-specific variants, while opting for Bonferroni over FDR to prioritize specificity over sensitivity given the breed’s complex genetic background and our focus on common variants (MAF>1%). We identified 145 SNPs surpassing the genome-wide significance thresholds. These significant SNPs exhibited additive effect sizes (Beta) ranging from 0.18 to 0.39, with larger Betas (e.g., 0.39 for the chr4:75645179 SNP associated with keel length) indicating a greater impact of the minor allele on trait variation (Tam et al., 2019). Such effect sizes align with the polygenic architecture of growth traits and complement the high cumulative SNP-based heritability (0.41∼0.86) observed in our study, underscoring that collective additive effects of these variants drive the strong genetic determination of growth traits in Baicheng You Chicken. Within the 200 kb flanking regions of these SNPs, 22 candidate genes were annotated, including *TAPT1, IGF2BP1, ADGRB3, LDB2, NCAPG*, and *LCORL*, which are likely associated with the genetic regulation of growth traits in this breed.

*TAPT1* is a key regulator of vertebrate axial skeletal patterning and ciliogenesis. Its loss of function can lead to skeletal homeotic transformations similar to those caused by *HOXC8* deficiency. *TAPT1* supports embryonic osteomyogenic differentiation through multiple mechanisms: it acts as a downstream effector of *HOXC8* to transduce extracellular patterning signals (Howell et al., 2007), maintains Golgi-cilium axis protein trafficking homeostasis to ensure proper ciliogenesis (Yusifov et al., 2021), and negatively regulates BMP pathway activity by controlling *SMAD1* protein stability, thereby precisely setting the threshold for osteogenic differentiation (Wang et al., 2022). In this study, *TAPT1* was identified as a candidate gene associated with chest depth (CD) and keel length (KL) in Baicheng You chicken. This finding aligns with the identification of *TAPT1* as a candidate gene for shank circumference and body slope length in Wenchang chicken GWAS (Luo et al., 2024), providing cross-breed genetic evidence for its conserved role in regulating growth traits in chickens. GO enrichment analysis further indicated that *TAPT1* is significantly enriched in the "cilium assembly" process (GO:0042384) under the "cell projection organization" pathway. This pathway is critically linked to growth phenotypes: *TAPT1* protein localizes to the centrosome/ciliary base, and its dysfunction disrupts ciliogenesis, subsequently interfering with key developmental signaling pathways like Hedgehog/BMP, leading to severe skeletal maldevelopment and growth retardation (Gene Ontology Consortium, 2021; Symoens et al., 2015). Previous research has confirmed that cilia-mediated Hedgehog signaling is essential for skeletal development in chicken embryos, and ciliary defects cause axial skeletal abnormalities (Davey et al., 2006). Our study revealed a high genetic correlation (0.95) between body slope length and keel length. We speculate that the *TAPT1* genetic variants identified in Baicheng You chicken (e.g., potential missense mutations affecting SMAD1/5 stability) may fine-tune the activation threshold of the BMP pathway. By moderately regulating the synthesis rate of collagen I, these variants could establish a distinct skeletal developmental pattern compared to commercial broiler breeds.

*IGF2BP1* is an m6A reader RNA-binding protein that plays a central role in maintaining the balance between myoblast proliferation and differentiation. It functions by recognizing and stabilizing target mRNAs such as *IGF2, FGFR1*, and *MYOG/MYH2*, primarily promoting cell survival during proliferation while preventing premature differentiation (Liu et al., 2024). Poultry research has provided multi-dimensional evidence supporting its role in growth regulation: chicken pan-genome analysis revealed associations between its promoter L1/L2 structural variations and growth/body size traits (Wang et al., 2021); Longsheng Feng chicken studies confirmed its functional activity in myoblasts (Li et al., 2024); and large-scale whole-genome sequencing established it as a key candidate gene for breast muscle weight in local chicken breeds (Tan et al., 2021), *collective evidence identifies IGF2BP1 as a known functional locus*. In this study, the "cell projection organization" pathway associated with *IGF2BP1* was further refined to "myoblast filopodia formation" (GO:0030030), a prerequisite for myotube fusion. *IGF2BP1* may upregulate N-cadherin expression by stabilizing *MYOD1/IGF2* mRNA, and knockdown of N-cadherin in chicken myoblasts has been shown to reduce filopodia formation and impair fusion efficiency (Zhang et al., 2020). This regulatory axis suggests a developmental shift toward investing resources in the refined architecture of muscle fibers rather than mere quantitative accumulation. Consequently, we propose that the "moderate expression level" of *IGF2BP1* reflects a quality-oriented muscle development strategy: it ensures fundamental growth while optimizing myofiber quality, thereby providing the cellular basis for forming finer-textured, superior-quality meat.

*ADGRB3* belongs to the adhesion G protein-coupled receptor (GPCR) family. Its canonical functions involve acting as a cell surface receptor regulating synaptogenesis, dendritic development, and myoblast fusion, rather than directly mediating lipolysis (Lanoue et al., 2013). Recent studies have shown that *ADGRB3* deficiency leads to abnormal energy expenditure in mice, suggesting a potential indirect regulatory role in systemic energy balance (Alsharif et al., 2023). In poultry metabolism research, integrative meta-GWAS and lipidomics have identified multiple QTLs associated with abdominal fat percentage and causal lipid molecules, indicating that lipid metabolism genes may indirectly influence growth by modulating "energy allocation" efficiency (An et al., 2026). In this study, the chr3:83455455 SNP within the *ADGRB3* locus was significantly associated with body weight (BW) and body slope length (BSL). Based on this, we hypothesize that this gene likely influences energy allocation not through classical lipolytic pathways, but by regulating the efficiency of myoblast fusion. Increased *ADGRB3* activity may enhance skeletal muscle formation efficiency, thereby directing more energy toward the "muscle-skeleton" growth axis rather than promoting excessive abdominal fat deposition. This proposed mechanism of optimized energy allocation aligns well with the "roughage tolerance" trait—characterized by high feed utilization efficiency—observed in Baicheng You chicken.

A key finding of this study is the identification of overlapping quantitative trait loci (QTL) within the 75.2∼75.9 Mb region of GGA4. This region harbors three functionally important genes: *LDB2* (a transcriptional co-activator/co-repressor), *NCAPG* (a condensin complex subunit), and *LCORL* (a nuclear receptor corepressor). Significant SNPs in this region were associated with six growth traits—body weight, body slope length, keel length, chest circumference, hip width, and shank length-directly explaining the high genetic correlation (0.95) between keel length and body slope length. This multi-trait association provides genetic evidence for pleiotropy. *LDB2* acts as a scaffold protein that, through its LIM domain, assembles transcriptional complexes with cofactors such as *LMO2*, thereby regulating the expression of terminal myogenic differentiation genes like *MYOG* and myogenic factors such as *MEF2C*, playing a critical role in myocyte differentiation (Deane et al., 2003). In this study, the SNP 4:75645179 in *LDB2* was significantly associated with body weight, body slope length, and keel length. GO enrichment analysis indicated that *LDB2* is primarily involved in the biological process of "cell adhesion," specifically "N-cadherin-mediated myoblast adhesion" (GO:0007155). This result aligns with findings from Wenchang chicken GWAS, where *LDB2* was also identified as a candidate gene for growth traits (Cai et al., 2024). *NCAPG* and *LCORL* form a classic QTL cluster for body size regulation in chickens (Larkina et al., 2021). Previous research has shown that the 4:75903135 locus within the *NCAPG-LCORL* intronic region can be used for selecting body weight at first egg (Feng et al., 2024), providing cross-breed functional validation for the associations observed in this study. However, Baicheng You Chicken exhibits distinct characteristics, notably lower frequencies of favorable alleles (e.g., MAF < 0.4 for *LCORL* 4:75302291, compared to > 0.5 in commercial broilers). Integrating insights from 3D genomics on long-range gene regulation (Shen et al., 2024), we hypothesize that the unique haplotype in the *NCAPG-LCORL* region of Baicheng You Chicken may fine-tune the pace of cell proliferation, thereby modulating skeletal growth at a rate optimized for overall energy metabolism and developmental quality. This mechanism likely synergizes with the *LDB2*-mediated myo-osteogenic differentiation pathway, forming a homeostatic regulatory network that ensures coordinated development and trait quality. This integrated network may represent a developmental strategy—shaped by long-term natural and artificial selection—that balances growth efficiency with survival adaptability in this breed.

Therefore, the significantly associated SNPs and candidate genes (e.g., loci in *IGF2BP1, LCORL*, and *NCAPG*) identified in this study provide concrete molecular targets. These findings establish a foundation for developing marker-assisted selection (MAS) panels aimed at improving growth traits in Baicheng You Chicken, while strategically maintaining its genetic diversity and other valuable indigenous characteristics within conservation and breeding programs. Critically, the high genetic correlations (e.g., 0.95 between BSL and KL) and pleiotropic QTL regions (e.g., GGA4:75.2∼75.9 Mb spanning *LDB2/NCAPG/LCORL*) enable multi-trait MAS, allowing simultaneous selection for body weight, body length, and keel length—maximizing genetic gain while minimizing the loss of breed-specific traits like meat quality and stress tolerance. Nevertheless, this study has several limitations that should be considered. First, the functional causality of the prioritized candidate genes and variants requires experimental validation through in vitro (e.g., gene editing in chicken cells) and in vivo (e.g., knock-in/knockout models) approaches. Second, the moderate sequencing depth (∼5x) and the specific 18-week sampling time point may not have captured all rare variants or dynamic expression patterns influencing growth across different developmental stages. Future work involving deeper sequencing, longitudinal sampling, and multi-omics integration (e.g., transcriptomics, proteomics of key tissues) will be essential to elucidate the precise regulatory mechanisms. Finally, the predicted genetic gains from MAS strategies need to be empirically tested in commercial or conservation farming environments to assess their practical effectiveness and any potential unintended effects on other economically important traits. Despite these limitations, the genetic architecture and molecular targets revealed here offer a robust scientific basis for the sustainable genetic improvement and conservation of the Baicheng You Chicken.

## Conclusion

This study provides the first systematic dissection of the genetic basis of growth traits in Baicheng You Chicken, an unimproved indigenous breed, through whole-genome resequencing-based GWAS, statistical fine-mapping, and genetic parameter estimation. The results revealed a polygenic architecture dominated by pleiotropic major QTLs. Eight growth traits exhibited moderate-to-high SNP-based heritability (0.41∼0.86) and high genetic correlations (0.76∼0.96), indicating strong potential for multi-trait coordinated selection. Analysis of 1,535 individuals identified 145 genome-wide significant SNPs, with fine-mapping prioritizing 22 high-confidence candidate genes. Among these, *TAPT1, IGF2BP1, ADGRB3, LDB2, NCAPG*, and *LCORL* emerged as core candidates regulating myogenesis, osteogenesis, energy metabolism, and cell proliferation. Colocalization analysis confirmed the causal role of variants in the GGA4:75.2∼75.9 Mb region. These findings have direct applications in breeding: validated SNPs in genes like *LDB2* and *NCAPG* can be used to develop low-density chips for marker-assisted selection to improve growth rate while preserving meat quality; the 2.02 million high-quality SNPs and high genetic correlations enable accurate multi-trait genomic selection; and allele frequency maps provide a basis for introgressing favorable growth alleles while maintaining genetic diversity and adaptive traits such as disease resistance. Future work should involve functional validation of candidate variants through cellular assays, tissue-specific eQTL analyses to elucidate regulatory mechanisms, and field testing of MAS panels to quantify actual genetic gains. In summary, this study elucidates the genetic mechanisms underlying growth in Baicheng You Chicken and establishes a scientific framework with actionable molecular targets for enhancing productivity and ensuring the sustainable conservation and utilization of this valuable genetic resource.

## CRediT authorship contribution statement

**Tinghao Jiang:** Writing – original draft, Validation, Methodology, Investigation, Formal analysis, Conceptualization. **Gaoyun You:** Writing – original draft, Validation, Methodology, Investigation, Formal analysis, Conceptualization. **Haiying Li:** Writing – review & editing, Supervision, Resources, Project administration, Funding acquisition, Conceptualization. **Zhuocheng Hou:** Validation, Investigation, Data curation. **Xiaoyu Zhao:** Visualization, Software, Formal analysis. **Wei Dong:** Writing – review & editing, Resources. **Herong Liao:** Resources, Project administration. **Shihao Zhang:** Validation, Software, Investigation. **Zhongtao Yin:** Writing – review & editing, Supervision, Resources, Project administration, Funding acquisition, Conceptualization.

## Disclosures

The authors declare the following potential financial and/or Homo sapiens conflicts of interest.

•Non-financial interests:

Author [TingHao Jiang] is a student at [Xinjiang Agricultural University].

•Conflict-free statement:

All authors declare that there are no financial or Homo sapiens conflicts of interest that could be construed as influencing the results of this study. We confirm that the above disclosures cover all potential conflicts of interest identified by all authors.

## Footnotes

Supplementary material associated with this article can be found, in the online version, at doi:10.1016/j.psj.2026.107063.
