# Supplementary material for: Mining of important genetic loci and evaluation of genetic effects for growth traits in Baicheng You Chicken
Source: Poult Sci. 2026 May 1;105(11):107063. doi: 10.1016/j.psj.2026.107063 (PMC13590092; doi:10.1016/j.psj.2026.107063)
Supplement: Supplementary file 1 [file mmc1.pdf]

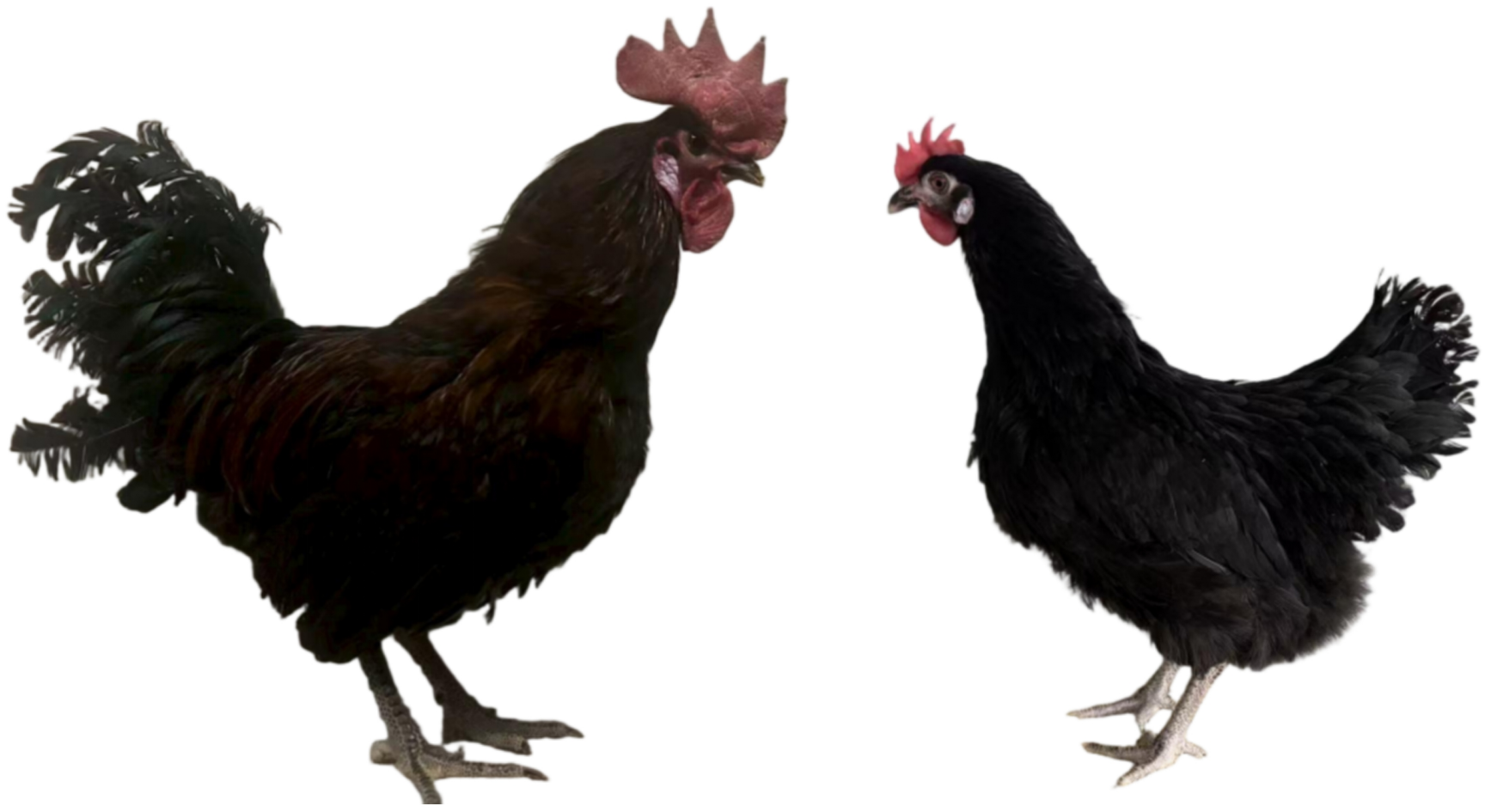

A B

Supplementary Figure 1. Representative photographs of Baicheng You Chicken. Panel A shows the lateral view of an adult rooster, and Panel B shows the lateral view of an adult hen.

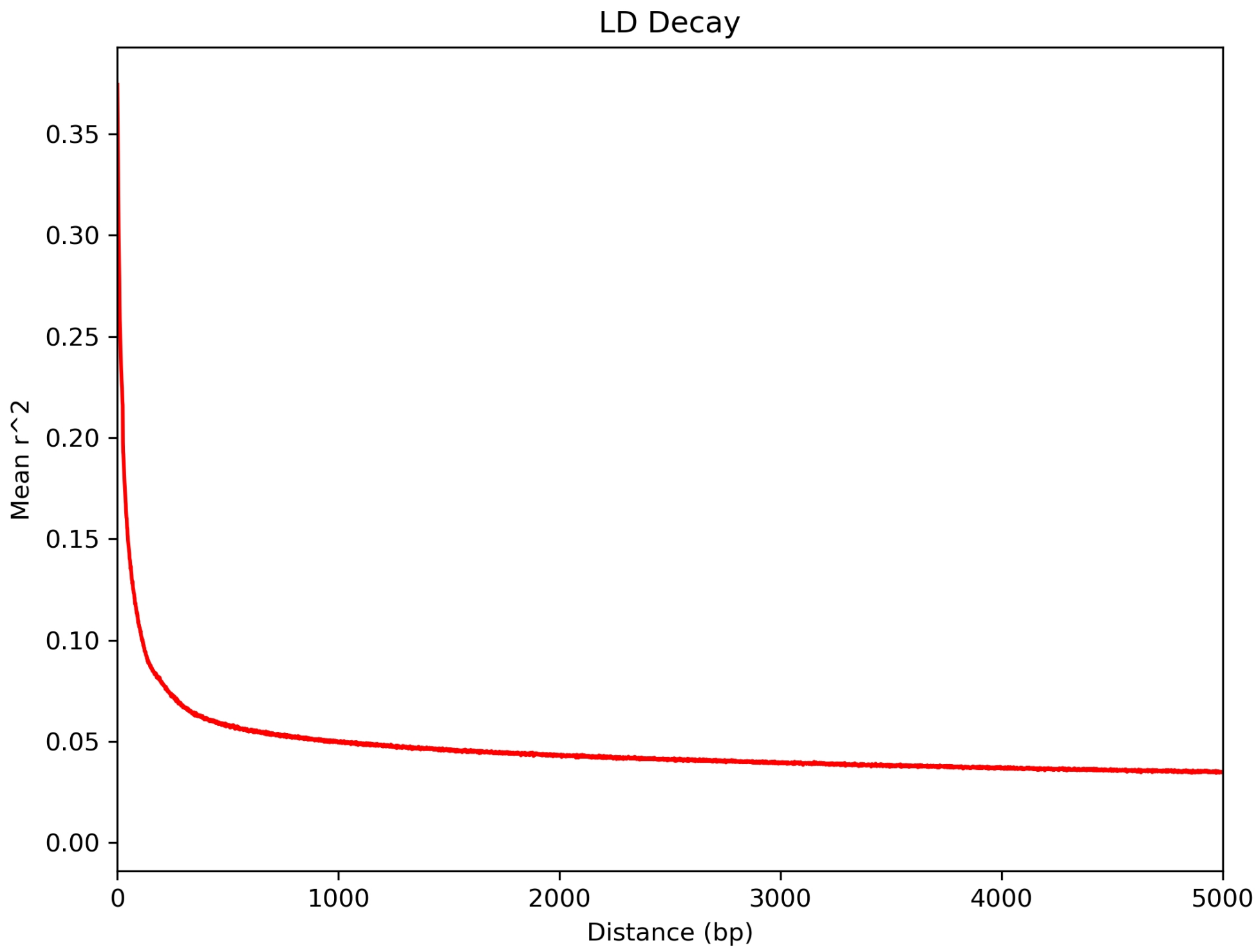

Supplementary Figure 2. Linkage disequilibrium (r^2) decay curve of the Baicheng You chicken population. The x-axis represents the physical distance between SNPs (0-5000 bp), and the y-axis represents the average linkage disequilibrium coefficient r^2. In this study, the LD decay rate of the Baicheng You chicken population was relatively rapid, with r^2 decaying to 0.2 at approximately 100 bp and to 0.1 at around 300 bp. By 5000 bp, r^2 had decreased to ~0.04, reaching a plateau phase.

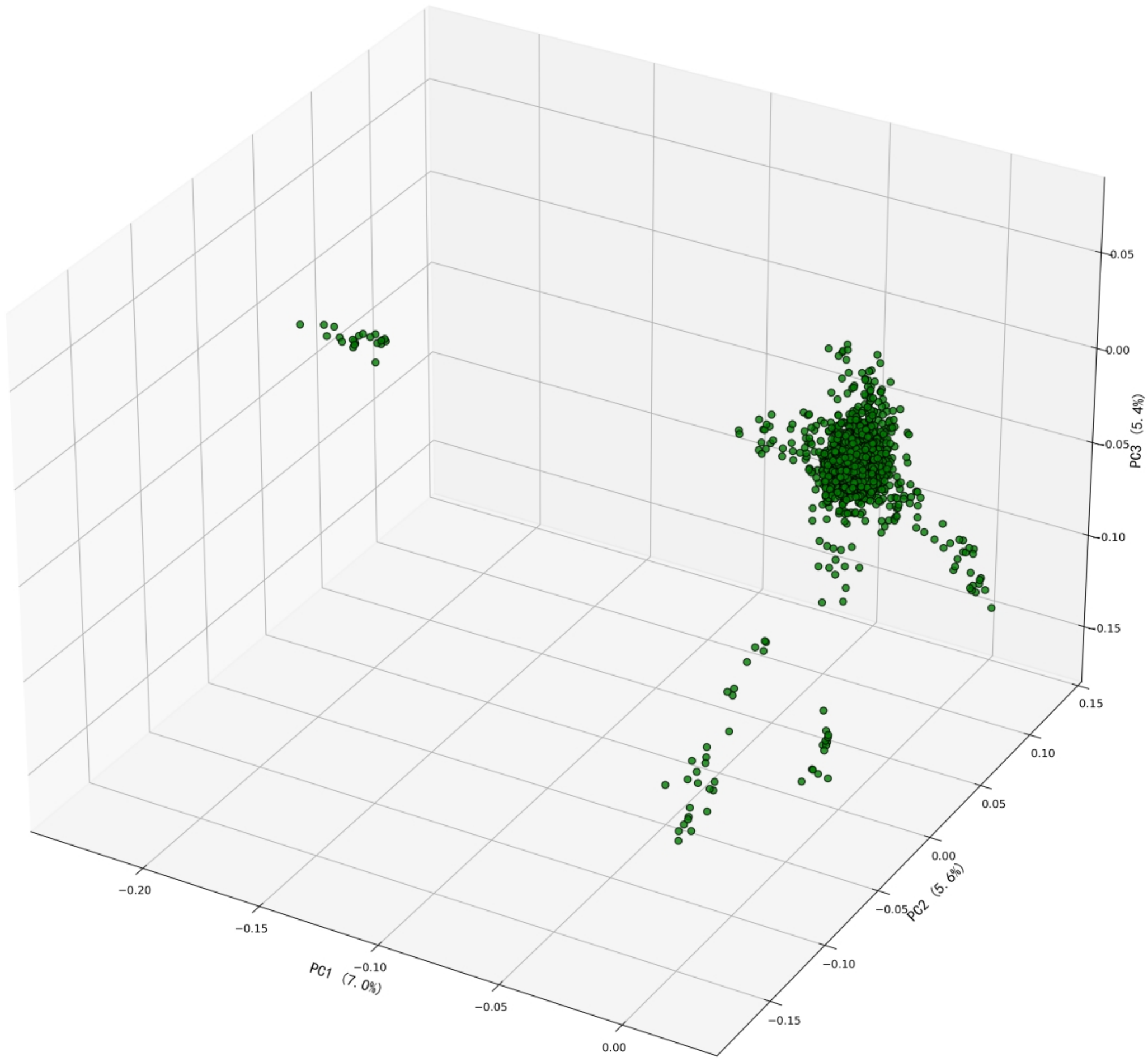

Supplementary Figure 3. Principal component analysis (PCA) of 1535(180 males and 1,355 females) Baicheng You chickens based on genome-wide autosomal SNPs. The 3D scatter plot shows the distribution of individuals along the first three principal components (PC1, PC2, and PC3). The percentages on the axes indicate the proportion of total genetic variance explained by each component. The analysis confirms the absence of significant population stratification within the flock, validating its suitability for genome-wide association analysis.

A

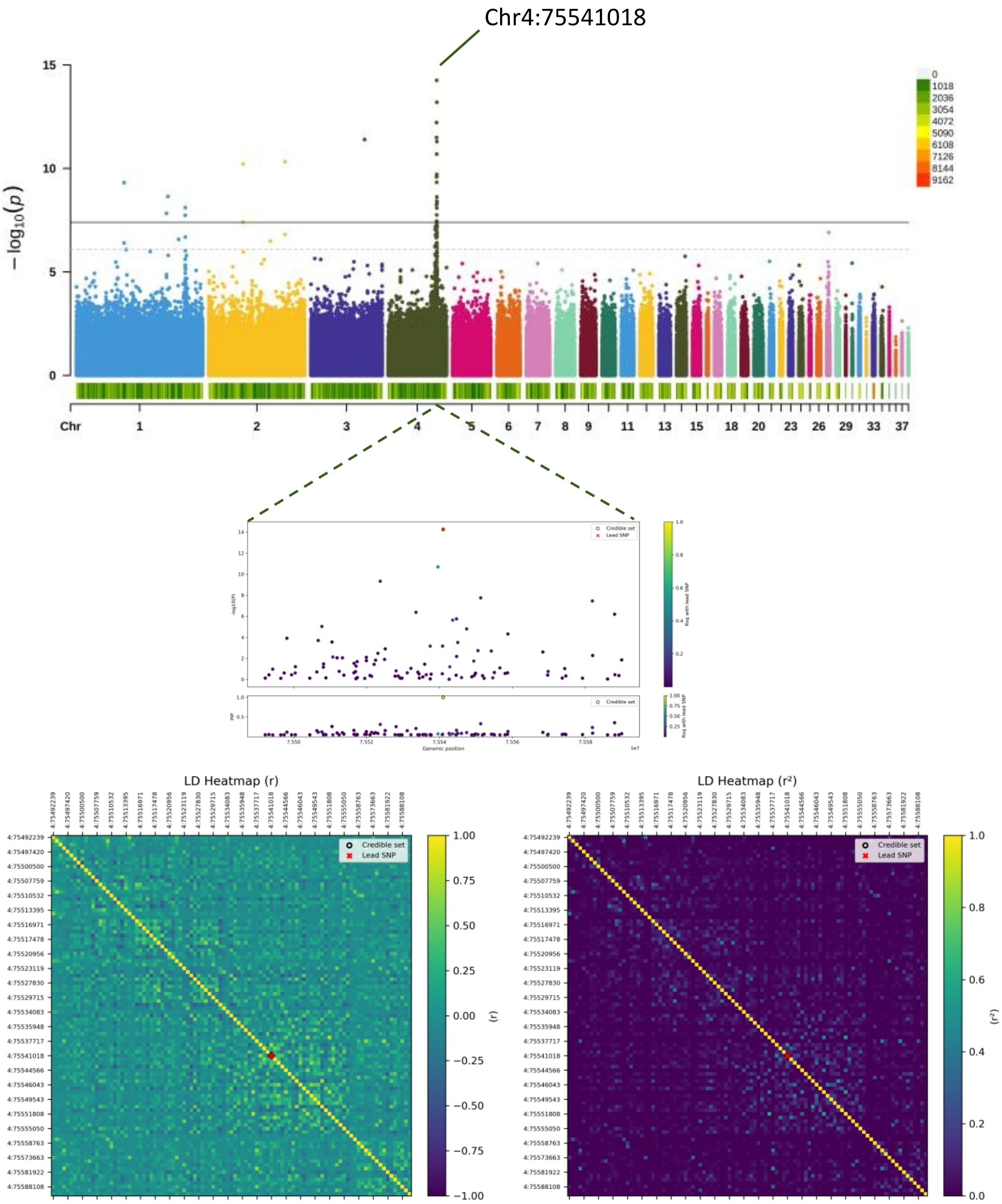
Chr4:75541018
15
10
5
0
$-\log_{10}(p)$
0
1018
2036
3054
4072
5090
6108
7126
8144
9162
Chr
1
2
3
4
5
6
7
8
9
11
13
15
18
20
23
26
29
33
37
Credible set
Lead SNP
LD Heatmap (r)
LD Heatmap (r²)
4:75492239
4:75497420
4:75500500
4:75507759
4:75510532
4:75513395
4:75516971
4:75517478
4:75520956
4:75523119
4:75527830
4:75529715
4:75534083
4:75535948
4:75537717
4:75541018
4:75544566
4:75546043
4:75549543
4:75551808
4:75555050
4:75558763
4:75573663
4:75581922
4:75588108
1.00
0.75
0.50
0.25
0.00
−0.25
−0.50
−0.75
−1.00
(r)
1.0
0.8
0.6
0.4
0.2
0.0
(r²)

B

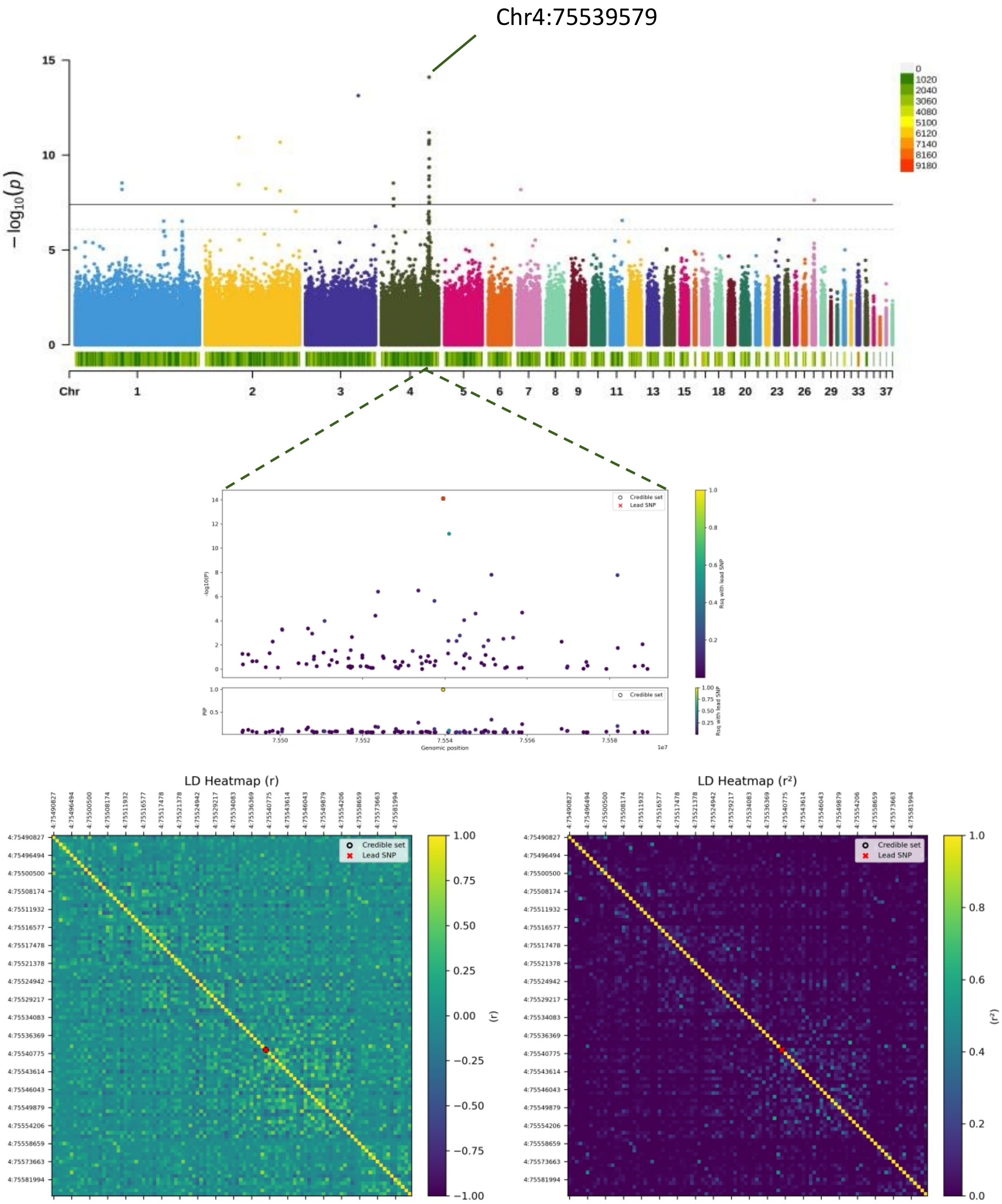

Chr4:75539579
$-\log_{10}(p)$
15
10
5
0
Chr
1
2
3
4
5
6
7
8
9
11
13
15
18
20
23
26
29
33
37
0
1020
2040
3060
4080
5100
6120
7140
8160
9180
Credible set
Lead SNP
Genomic position
LD Heatmap (r)
LD Heatmap (r²)
4:75490827
4:75496494
4:75500500
4:75508174
4:75511932
4:75516577
4:75517478
4:75521378
4:75524942
4:75529217
4:75534083
4:75536369
4:75540775
4:75543614
4:75546043
4:75549879
4:75554206
4:75558659
4:75573663
4:75581994

C

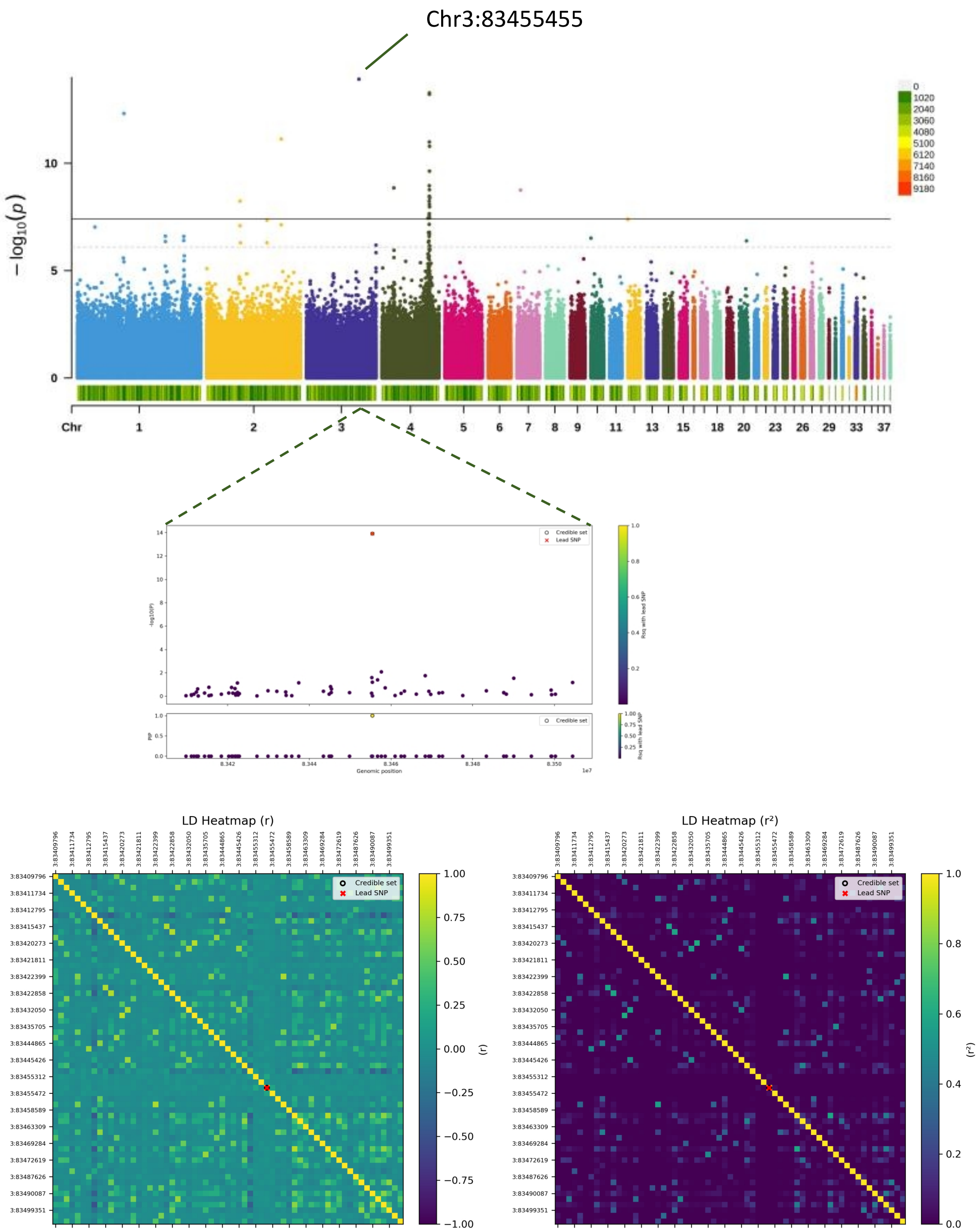

Chr3:83455455
$-\log_{10}(p)$
Chr
LD Heatmap (r)
LD Heatmap (r²)
Credible set
Lead SNP
(r)
(r²)

D

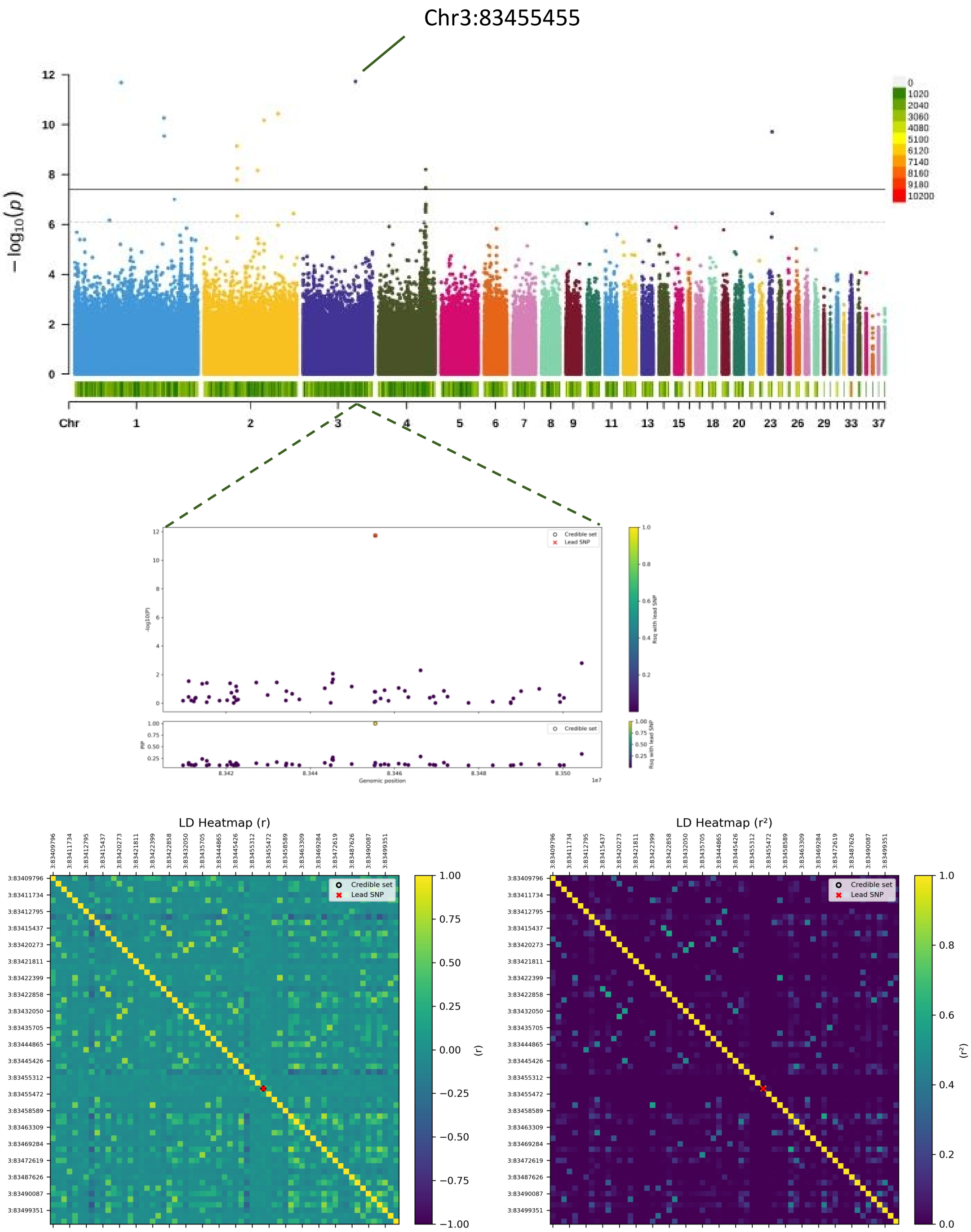

Chr3:83455455
$-\log_{10}(p)$
Chr
1
2
3
4
5
6
7
8
9
11
13
15
18
20
23
26
29
33
37
0
1020
2040
3060
4080
5100
6120
7140
8160
9180
10200
Credible set
Lead SNP
Genomic position
LD Heatmap (r)
LD Heatmap (r²)
(r)
(r²)

E

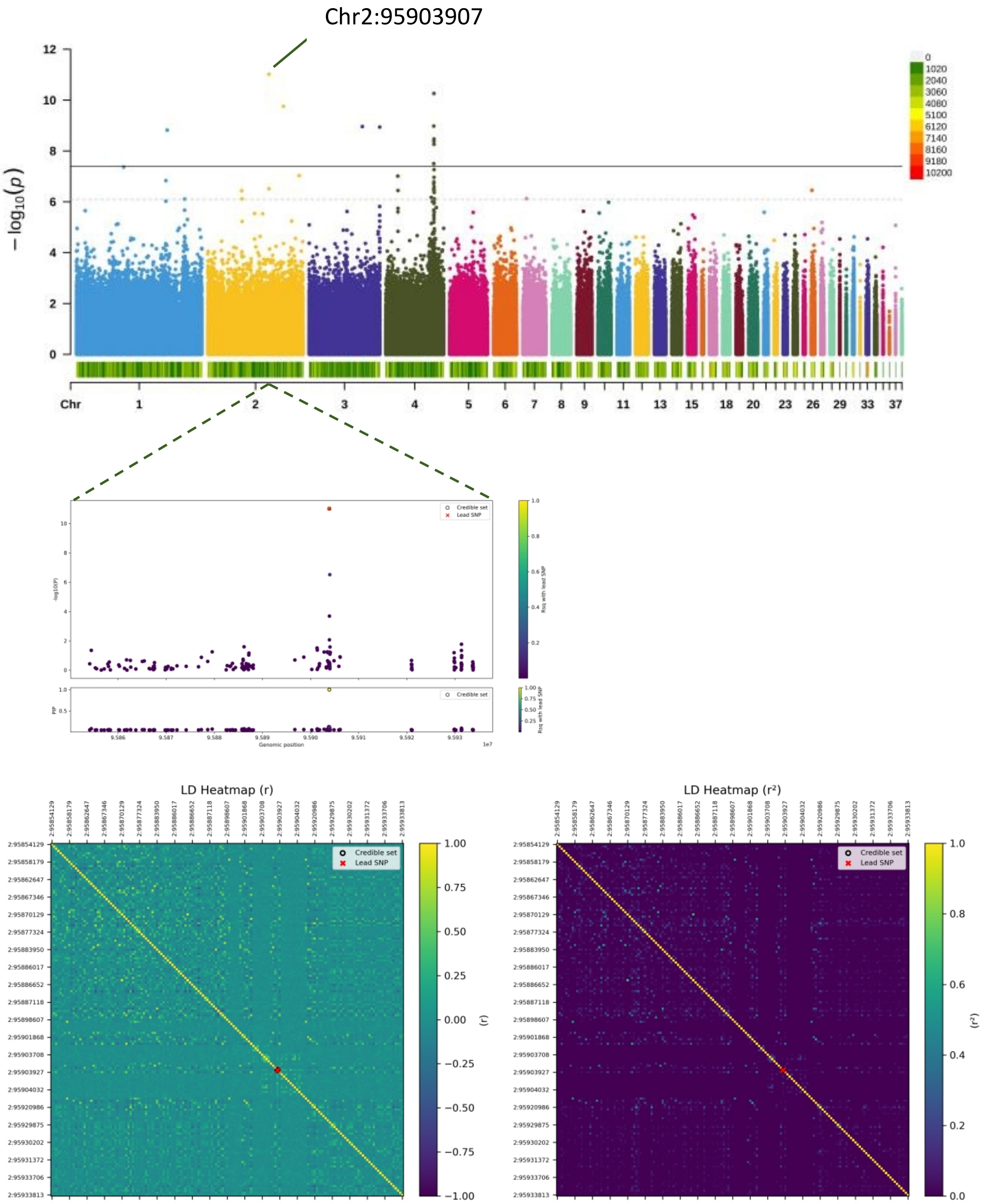

Chr2:95903907
$-\log_{10}(p)$
Chr
Credible set
Lead SNP
Genomic position
LD Heatmap (r)
LD Heatmap (r²)

F

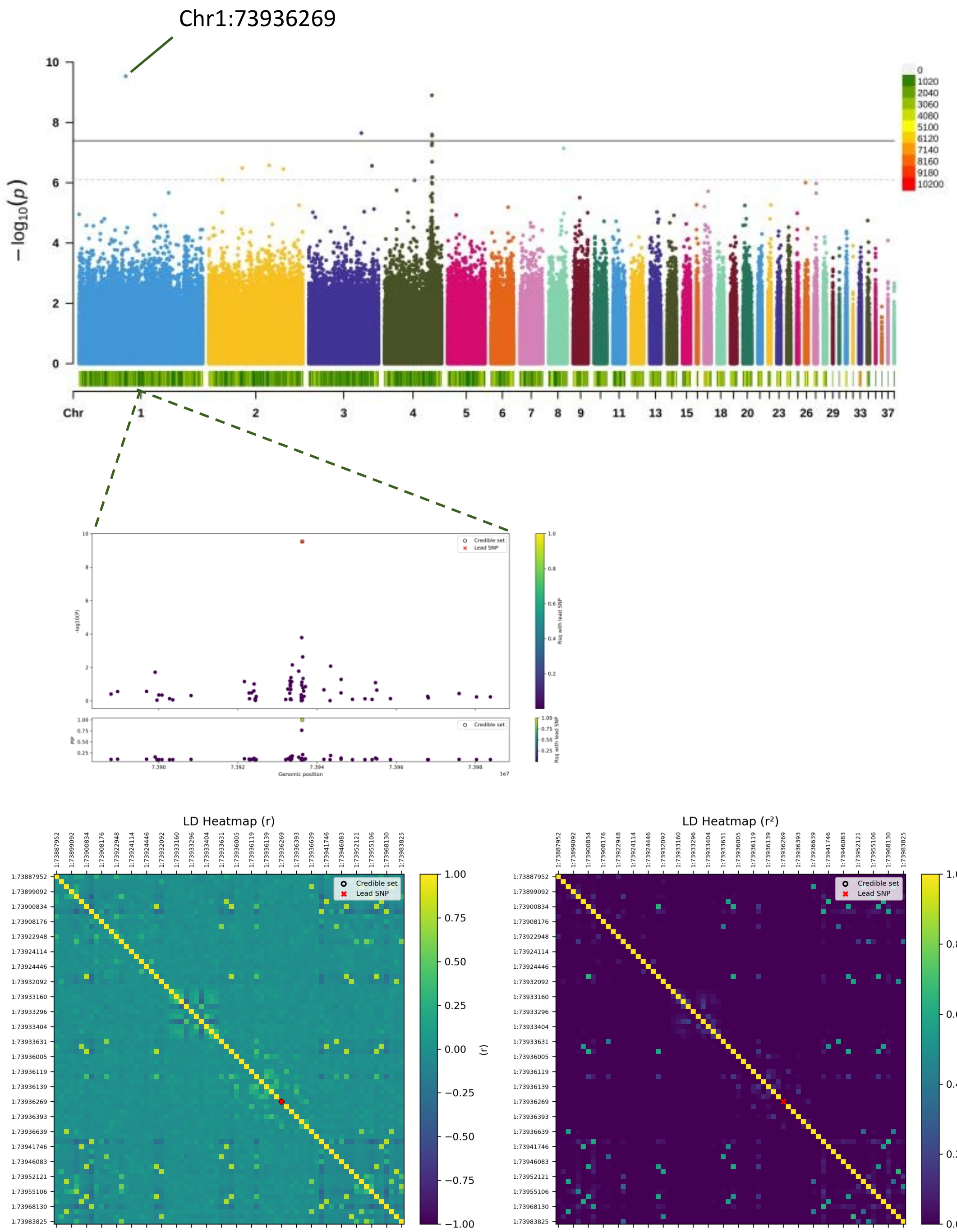

Chr1:73936269
$-\log_{10}(p)$
Chr
1
2
3
4
5
6
7
8
9
11
13
15
18
20
23
26
29
33
37
0
1020
2040
3060
4080
5100
6120
7140
8160
9180
10200
Credible set
Lead SNP
Genomic position
LD Heatmap (r)
LD Heatmap (r²)
(r)
(r²)
1:73887952
1:73899092
1:73900834
1:73908176
1:73922948
1:73924114
1:73924446
1:73932092
1:73933160
1:73933296
1:73933404
1:73933631
1:73936005
1:73936119
1:73936139
1:73936269
1:73936393
1:73936639
1:73941746
1:73946083
1:73952121
1:73955106
1:73968130
1:73983825

G

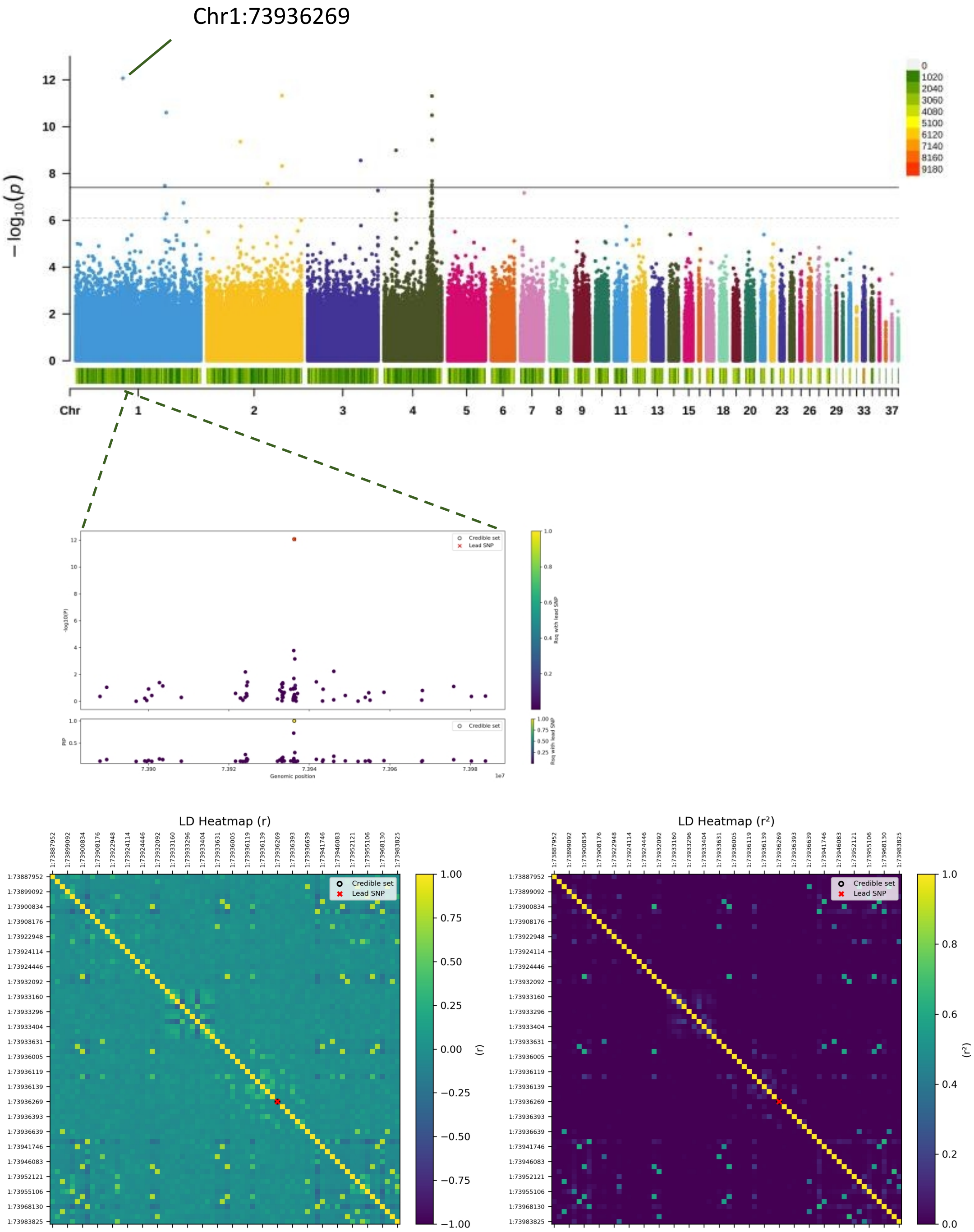

Chr1:73936269
−log10(p)
12
10
8
6
4
2
0
0
1020
2040
3060
4080
5100
6120
7140
8160
9180
Chr
1
2
3
4
5
6
7
8
9
11
13
15
18
20
23
26
29
33
37
Credible set
Lead SNP
Genomic position
LD Heatmap (r)
LD Heatmap (r²)
1:73887952
1:73899092
1:73900834
1:73908176
1:73922948
1:73924114
1:73924446
1:73932092
1:73933160
1:73933296
1:73933404
1:73933631
1:73936005
1:73936119
1:73936139
1:73936269
1:73936393
1:73936639
1:73941746
1:73946083
1:73952121
1:73955106
1:73968130
1:73983825
1.00
0.75
0.50
0.25
0.00
−0.25
−0.50
−0.75
−1.00
(r)
1.0
0.8
0.6
0.4
0.2
0.0
(r²)

H

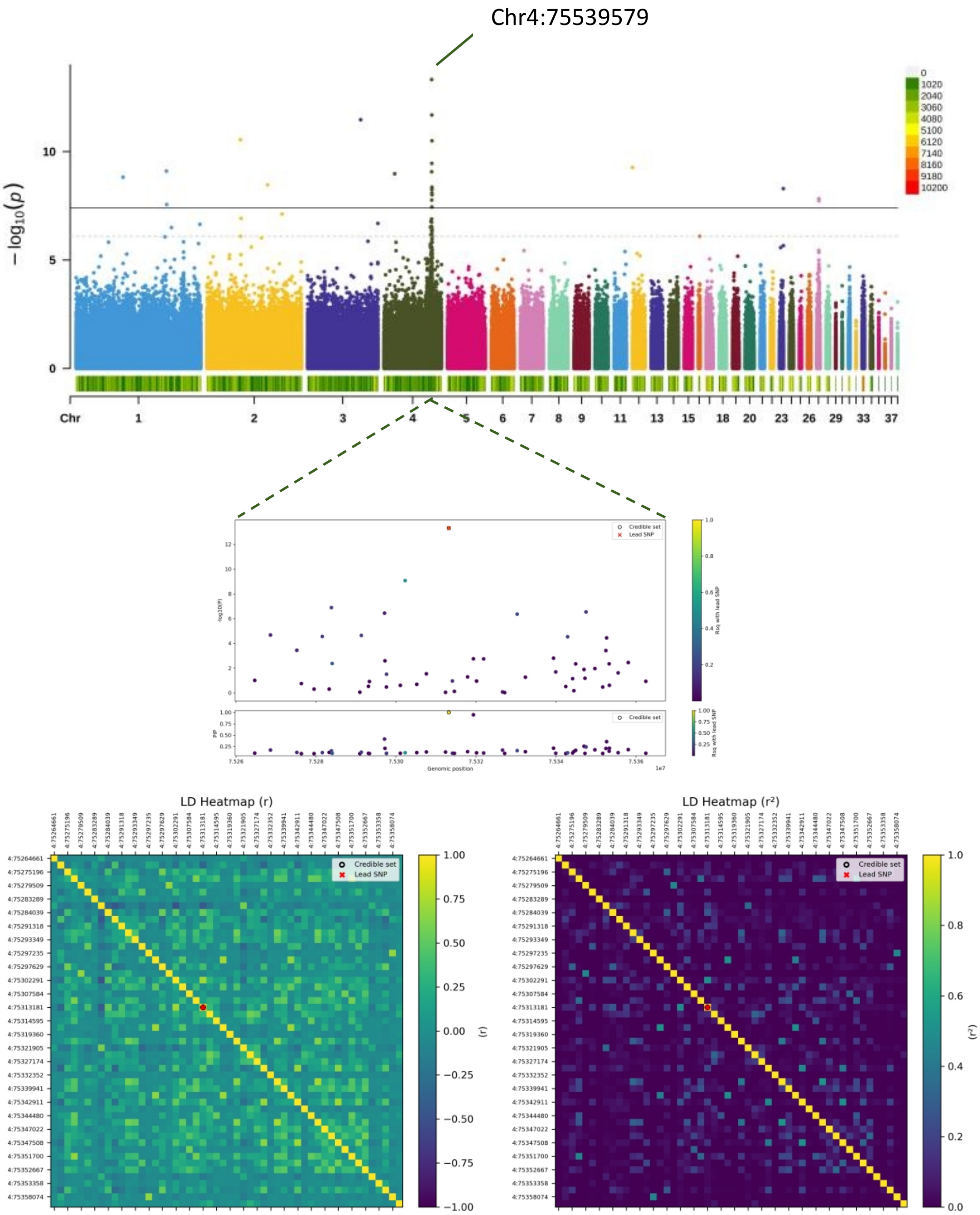

Supplementary Figure 4. Statistical fine-mapping and linkage disequilibrium (LD) analysis of genome-wide significant loci.(A) For the body weight (BW) locus on chromosome GGA4. (B) For the body slanting length (BSL) locus on chromosome GGA4. (C) For the keel length (KL) locus on chromosome GGA3. (D) For the chest girth (CG) locus on chromosome GGA3. (E) For the pelvic width (PW) locus on chromosome GGA2. (F) For the chest width (CW) locus on chromosome GGA1. (G) For the chest depth (CD) locus on chromosome GGA1. (H) For the shank length (SL)

locus on chromosome GGA4. For each locus, the upper panel is a regional Manhattan plot showing association signals; the middle panel displays the posterior inclusion probability (PIP) of each variant from the fine-mapping analysis (SuSiE), highlighting the lead single nucleotide polymorphism (SNP) and its 95% credible interval; the lower panel is an LD heatmap (measured by r^2) relative to the lead SNP (indicated by a red arrow), illustrating the linkage structure of the region. All genomic coordinates are based on the GRCg7b assembly.
